# Migratory responses in enucleated cells: The forces driving the locomotion movement of unicellular organisms

**DOI:** 10.1093/pnasnexus/pgaf232

**Published:** 2025-07-25

**Authors:** Ildefonso M De la Fuente, Jose Carrasco-Pujante, Maria Fedetz, Carlos Bringas, Alberto Pérez-Samartín, Gorka Pérez-Yarza, Luis Martínez, José I López, Jesus M Cortes, Iker Malaina

**Affiliations:** Department of Mathematics, Faculty of Science and Technology, University of the Basque Country, UPV/EHU, Barrio Sarriena s/n, Leioa 48940, Spain; Department of Nutrition, CEBAS-CSIC Institute, Espinardo University Campus, Murcia 30100, Spain; Department of Cell Biology and Histology, Faculty of Medicine and Nursing, University of the Basque Country, UPV/EHU, Barrio Sarriena s/n, Leioa 48940, Spain; Department of Cell Biology and Immunology, Institute of Parasitology and Biomedicine “López-Neyra”, CSIC, Avda. del Conocimiento 17, Armilla (Granada) 18016, Spain; Department of Cell Biology and Histology, Faculty of Medicine and Nursing, University of the Basque Country, UPV/EHU, Barrio Sarriena s/n, Leioa 48940, Spain; Department of Neurosciences, Faculty of Medicine and Nursing, University of the Basque Country, UPV/EHU, Barrio Sarriena s/n, Leioa 48940, Spain; Department of Cell Biology and Histology, Faculty of Medicine and Nursing, University of the Basque Country, UPV/EHU, Barrio Sarriena s/n, Leioa 48940, Spain; Department of Mathematics, Faculty of Science and Technology, University of the Basque Country, UPV/EHU, Barrio Sarriena s/n, Leioa 48940, Spain; Biobizkaia Health Research Institute, Plaza Cruces s/n, Barakaldo 48903, Spain; Department of Cell Biology and Histology, Faculty of Medicine and Nursing, University of the Basque Country, UPV/EHU, Barrio Sarriena s/n, Leioa 48940, Spain; Biobizkaia Health Research Institute, Plaza Cruces s/n, Barakaldo 48903, Spain; IKERBASQUE: The Basque Foundation for Science, Plaza Euskadi 5, Bilbao 48009, Spain; Department of Mathematics, Faculty of Science and Technology, University of the Basque Country, UPV/EHU, Barrio Sarriena s/n, Leioa 48940, Spain

**Keywords:** cellular migration, systemic behavior, galvanotaxis, chemotaxis, cytoplasts

## Abstract

Locomotion movements are a fundamental characteristic of a variety of species, including prokaryotic and eukaryotic, that has a high impact on essential physiological and pathological processes. For decades, many different authors have focused on studying specific individual processes and their corresponding biomolecular components involved in cellular locomotion movements. Recently, we have shown that locomotion movements are regulated by integrative self-organized molecular processes operating at the systemic level. Here, to verify that said systemic behavior also exists in extreme critical physiological conditions such as those corresponding to enucleated cells, we carried out an extensive study with 200 enucleated cells (cytoplasts) belonging to the *Amoeba proteus* species. The migratory movements of both enucleated and nonenucleated cells (400 in total) have been individually studied in four different scenarios: in the absence of stimuli, under a galvanotactic field, in a chemotactic gradient, and under complex conditions such as simultaneous galvanotactic and chemotactic stimuli. All the experimental trajectories were analyzed using nonlinear quantitative metrics for individual cell trajectories. The results show that both nonenucleated amoebas and cytoplasts display the same type of dynamic migratory patterns. The locomotion displacements of enucleated cells are a consequence of complex self-organized molecular dynamics, modulated at a systemic-cytoplasmic level. We have also quantitatively detected that enucleation clearly affects the correlation times and the intensity of the migratory responses of cytoplasts. The fact that cytoplasts preserved the dynamic properties of their migratory trajectories when compared with nonenucleated cells suggests that nuclear activity has a minor role in regulating the locomotion displacements of cells.

Significance Statement Cell migration is a fundamental process in a wide range of organisms, including prokaryotic and eukaryotic. The cellular nucleus contains DNA required for most protein synthesis in eukaryotes. Can a cell without a nucleus exhibit migratory behavior similar to that found in cells with a nucleus? To address this question, we have carefully removed the nucleus and exposed *Amoeba proteus* cells to different tests for migratory displacements. Our results indicate that both nonenucleated and enucleated amoebas display the same type of dynamic migratory patterns. The fact that enucleated amoebas preserved the dynamic properties of their locomotion trajectories as well as the nonenucleated cells suggests that nuclear activity has a minor role in regulating the systemic locomotion displacements of cells.

## Introduction

Cell migration is an extremely complex and tightly regulated process, by which cells belonging to a wide range of species, from prokaryotic to eukaryotic, move from one location to another within their external microenvironments. The adequate directional movements of cells are critical for life in all Metazoan organisms and play a central role in essential biological processes, such as embryogenesis (creation of the endodermal, mesodermal, and ectodermal layers during gastrulation), organogenesis, neural development, wound healing (e.g. somatic stem cell types for tissue repair, as well as mesenchymal and hematopoietic stem cells, display remarkable migratory capabilities, mobilizing to injury sites to participate in their regeneration), angiogenesis, and immune responses (1).

In humans, misguided cellular locomotion responses play a significant role in numerous pathologies, including atherosclerosis (2), the progression of metastatic tumors (3–6), progeria (7), osteoarthritis (8), fibrosis (9–11), auditory impairments (12), chronic obstructive pulmonary disease (13), immune-related actinopathies (14), endometriosis (15, 16), multiple sclerosis (17, 18), rheumatoid arthritis (19, 20), diabetic wound (21), psoriasis (22, 23), asthma (24–26), Crohn's disease (27, 28), congenital brain disorders (29–31), and other types of immunodeficiencies (32).

Free-living cells efficiently explore their external environment to find food, adjusting with precision their trajectory and speed while avoiding predators and adverse conditions. When unicellular organisms both prokaryotes and eukaryotes migrate, they need to integrate a variety of external cues to carry out efficient movements, maximize their displacement rate, and develop optimal search trajectories to localize their targets. These strategies become particularly critical when the cells lack enough information on where the target is located.

The important physiological processes in which cell migration is involved, as well as its related human pathologies, make this complex cellular behavior a crucial issue in contemporary biology. In fact, despite the many research efforts made, many aspects of cellular migration remain unknown as, for instance, how migratory cells coordinate the intracellular physiological processes, the subcellular organelles, and molecular-metabolic responses.

Recently, we showed that cellular locomotion movements depend on a complex integrated self-organized system, carefully regulated at the systemic level, which emerges from the cooperative nonlinear interaction between a large repertoire of cellular components. Such migratory property seems to be not found in partial mechanisms, in any specific molecular part, or individual physiological processes of the cell (33).

Supporting this thesis, various studies have shown that most of the cellular physiological processes are involved in cell migration. In addition to the main cytoskeletal components (actin microfilaments, microtubules, and intermediate filaments) energy is a vital factor in cell motility; the cytoskeleton converts chemical energy into mechanical forces requiring significant bioenergetic demands; hence, mitochondrial activity and the adenylate energy system (34) are key regulators of directional motion (35). The endoplasmic reticulum, a signaling organelle controlling multiple processes like calcium ions, also contributes to cell locomotion regulation (36, 37). Furthermore, cell polarity, influenced by centrosome positioning (38, 39) and centrosomal orientation (40, 41), is essential for directional motion. The Golgi apparatus, a key processing center for proteins modified in the endoplasmic reticulum, modulates intracellular traffic processes, influencing cell motility (42). The global nucleus structure acts as a central mechanosensory system, being crucial for appropriate mechanical responses during cell migration (43, 44). Structural chromatin organization also plays a significant role in cellular migration (45). In addition, numerous biochemical molecules and other physiological processes are involved in the directional movement of cells, including PI3K (46), focal adhesion proteins (47), actin-binding proteins (48), PLEKHG3 (49), p21-activated kinases (50), SCAR/WAVE proteins (51), Arp2/3 complexes (52), mitogen-activated protein kinases (53), actin-capping protein (54), WASP family proteins (55), phosphoinositide lipid molecules (56–58), TORC2/PKB pathway (59), and the Nck family of adaptor proteins (60), among others. These experimental evidences clearly indicate the existence of complex functional and integrative physiological-biomolecular processes involved in migratory behaviors at a cellular global scale.

Here, to continue the study of the dynamic forces driving the self-organized systemic behaviors that regulate the migratory movements (33), we have tested these integrative systemic properties in enucleated cells (cytoplasts), which represent extreme cellular physiological conditions. To carry out this aim, we have quantitatively analyzed the experimental trajectories of individual cytoplasts (enucleated by a Sutter MP-225 micromanipulator) belonging to the *Amoeba proteus* species using advanced tools and computational methods. Specifically, we have studied the essential characteristics of the locomotion movements in 200 cytoplasts, which were compared with 200 nonenucleated amoebas (400 single cells in total). All the trajectories have been analyzed using four advanced nonlinear physical–mathematical tools rooted in Statistical Physics, and three important kinematic properties.

The migratory behavior of both enucleated and nonenucleated cells has been individually studied in four different scenarios: in the absence of stimuli, under a galvanotactic field (the electric membrane potential of cells enables predators like amoebas to detect prey), in a chemotactic gradient (we have used an nFMLP peptide, which indicates to the amoebae the possible presence of food in their immediate environment), and under complex conditions such as simultaneous galvanotactic and chemotactic stimuli.

Note that, experimental evidence with *A. proteus* shows that these cells deprived of their nucleus through micromanipulation can continue to live for long periods, with an expectancy of up to 2 weeks (61). In fact, these studies have confirmed the complete viability of enucleated single-celled organisms after reimplantation of a new nucleus, 12 days elapsed since enucleation, whereupon some of these cells were able to fully resume their physiological functions, establishing viable cultures and performing adequate mitosis. It is noteworthy that the 12-day survival period of an enucleated amoeba far exceeds the typical 24-h cell cycle of an *A. proteus* under standard laboratory conditions (62).

Our quantitative analysis shows that both nonenucleated amoebas and cytoplasts display the same kind of complex dynamic structure of migratory movements. The fact that cytoplasts preserved the dynamic properties of their migratory trajectories when compared with nonenucleated cells suggests that the nuclear activity has a minor role in regulating the locomotion displacements of cells. In addition, we have observed that enucleation affects the correlation times and the intensity of the migratory response of cytoplasts.

We have quantitatively detected that enucleation clearly affects the correlation times and the intensity of the migratory responses of cytoplasts: we found that the average correlation time of the migratory responses (the time during which amoebal movement is influenced by previous steps) was shorter in cytoplasts (8.33/5.21 min) than in intact cells (10.42/8.33 min). The intensity of the migratory response, calculated as the module of the vector connecting the initial and final coordinates of each amoeba trajectory (displacement vector) was also smaller on average in enucleated cells (2.47/2.42 mm) than in intact cells (4.31/3.04 mm) (median/interquartile range [IQR] values, see Table 1).

**Table 1.** Comparison between the migratory properties of nonenucleated and enucleated *A. proteus* cells.

Quantitative tool	Nonenucleated (median/IQR)	Enucleated (median/IQR)	*P*-values (Wilcoxon rank-sum tests)
RMSF *α*	0.74/0.07	0.77/0.07	10^−11^
Correlation time (min)	10.42/8.33	8.33/5.21	0.001
MSD *β*	1.93/0.11	1.91/0.11	0.011
DFA *γ*	1.82/0.08	1.82/0.09	0.993
ApEn	0.002/0.001	0.003/0.001	10^−7^
Intensity of the response (mm)	4.31/3.04	2.47/2.42	10^−12^
Directionality ratio	0.62/0.21	0.60/0.28	0.047
Average speed (µm/s)	1.70/0.78	1.34/0.84	10^−8^

Numerical values (median/IQR) and Wilcoxon test *P*-values to evaluate inter-cell-type variability in kinematic and systemic properties.

The locomotion displacements of enucleated cells are regulated by emergent self-organized molecular dynamics, carefully regulated at a global-systemic level, which seems to depend on the cooperative nonlinear interaction of most of the molecular components of cytoplasm.

## Results

**Fig. 1.**
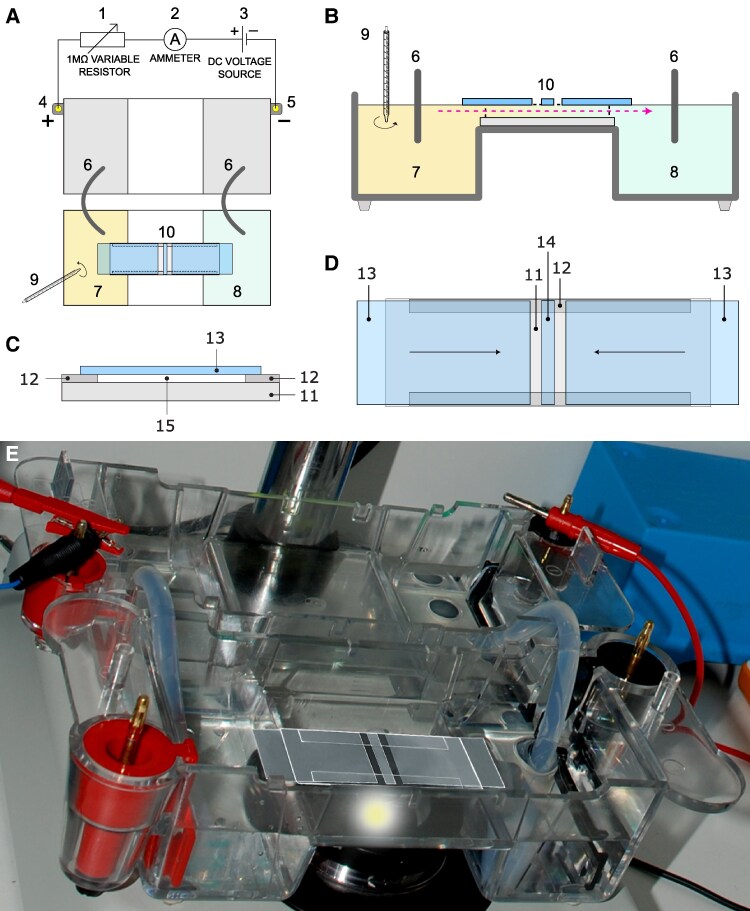
Experimental setup configuration. A) Top view of the experimental setup. B) A full section of the electrophoresis block where the amoebae were placed. 1: Variable resistor (1 MΩ) to adjust the amount of circulating electric current (intensity); 2: ammeter to keep track of electric current values over time; 3: BIO-RAD model 1000/500 power supply unit, configured to provide a stable DC voltage output; 4: positive electrode (anode); 5: negative electrode (cathode); 6: agar + KCl bridges; 7: well were the peptide was added; 8: well containing fresh Chalkley's simplified medium, toward which the chemical gradient spreads over time; 9: a stirrer was utilized for the adequate blending of the chemotactic peptide in the medium; 10: experimental glass chamber, the direction and trajectory of the laminar flow is indicated by a pink arrow. C) Sectional view of the experimental glass chamber. D) Top view of the experimental glass chamber. 11: standard glass slide; 12: two longitudinal 4 × 60 × 0.17 mm glass stripes glued to *11* with silicone adhesive; 13: lateral sliding glass pieces; 14: central 3 × 24 × 0.17 mm glass stripe where the amoebae were placed underneath. 15: cross-sectional area of the experimental glass chamber. See Materials and methods for more detail. E) Experimental setup under experimental conditions. The setup layout and its components, illustrated in A–D, as seen under experimental conditions. Panels A–D were adapted from Fig. 1 in https://academic.oup.com/pnasnexus/article/3/5/pgae171/7655426#461839591.

To perform all our experiments, we used a special setup consisting of two standard electrophoresis blocks (17.5 cm long), two agar bridges, a power supply, and a glass structure placed in the middle of the second electrophoresis block, where the amoebae were located (see Materials and methods and Fig. 1). We plugged one electrophoresis block into a power supply unit and connected the other one to it with two agar bridges. This way, the anode and cathode did not touch the medium (Chalkley's simplified medium (63)) where the amoebae swam. The glass structure made it possible to establish a laminar flux that let the electric current go through while also generating an nFMLP peptide gradient (see Materials and methods and Fig. 1).

Before conducting each experiment, the amoebae underwent a 24-h starvation period. We used a stereo microscope equipped with a digital camera to capture their individual migration patterns for 34′10″. To reduce the influence of social interactions among the amoebae, each replication consisted of small cell groups of size 2.63 ± 1.42 (mean ± SD) and never more than eight cells. The following basic experimental information data (BEID) parameters are provided for each scenario: “Nr” for the number of cells per replication, “Er” for the number of experimental replications, and “*N*” for the total number of cells. The recorded trajectories were then subjected to time series analysis using sophisticated nonlinear dynamic techniques.

**Fig. 2.**
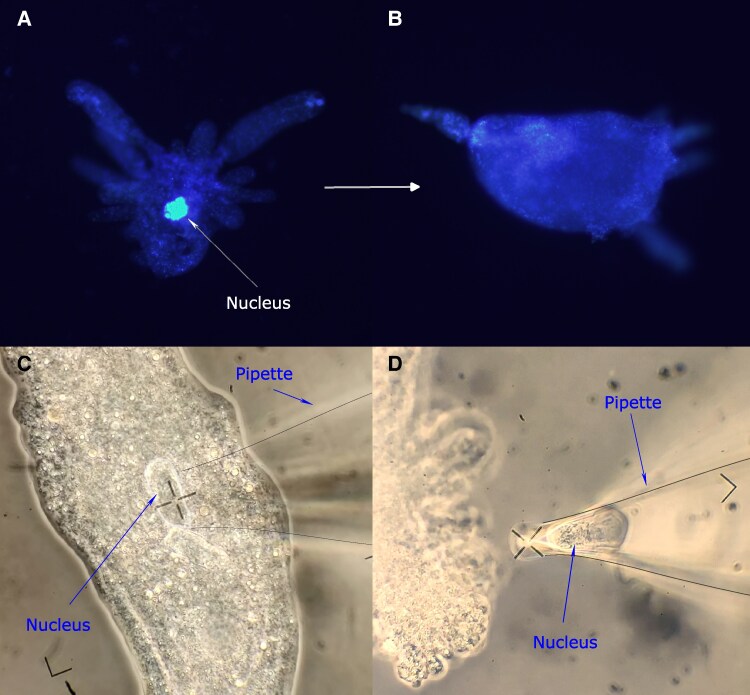
Cell enucleation procedure. A) A fluorescent microscopy image of *A. proteus* cell with its nucleus intact. B) A fluorescent microscopy image of an enucleated cell (cytoplast). A and B) Cytoplasts were stained with DAPI (1 μg/mL) after cell enucleation to confirm the absence of nucleus. C) Enucleation process 1: extracting the nucleus with a glass enucleation pipette. D) Enucleation process 2: enucleated cell (cytoplast), the extracted nucleus can be seen inside the enucleation pipette. See Materials and methods for more details. Enucleation pipettes in C and D have been outlined with a thin black stroke to enhance visualization.

The cells were enucleated using the “Sutter MP-225” computer-aided micromanipulator, which guarantees that the damage during enucleation is minimized, and left undisturbed until they exhibited an elongated shape with few thin pseudopodia and were firmly attached to the substrate, which took an average of ∼2 min. The enucleated cells were then placed in the experimental setup. Once they had regained the appropiate morphological and physiological characteristics (an elongated shape and strong attachment to substrate), the locomotion movements of each cytoplast were recorded. Lastly, the absence of the nucleus was verified by DAPI staining using an Olympus IX71 inverted fluorescence microscope (see Materials and methods and Fig. 2A and B).

We recorded and analyzed the migratory movements of 400 amoebae (200 cells and 200 cytoplasts) belonging to the species *A. proteus* under four experimental scenarios: without any stimulus, within an electric field, within a chemical gradient, and under simultaneous chemical and electrical stimuli.

### Cell motility in the absence of external cues

**Fig. 3.**
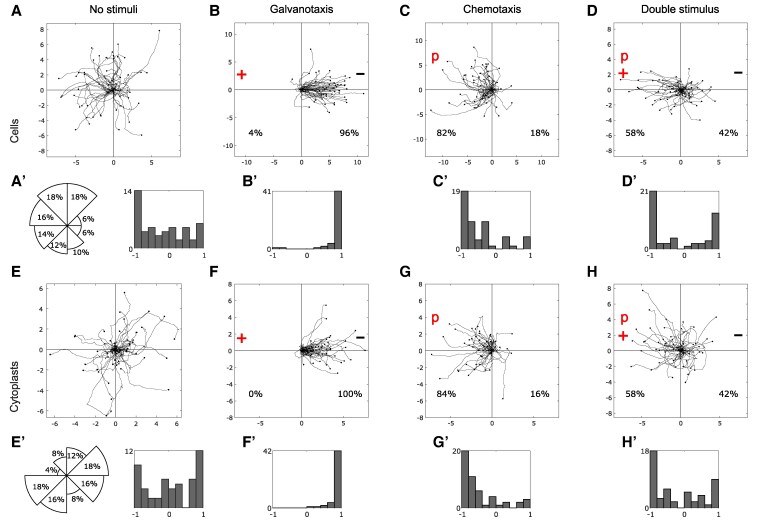
Cell and cytoplast motion under four experimental scenarios. A–H) Migratory trajectories of *A. proteus* cells (upper row) and cytoplasts (lower row) under four different experimental scenarios (*No stimuli*, *Galvanotaxis*, *Chemotaxis* and *Double stimulus*). Direction of cell (A′, left panel) and cytoplast (E′, left panel) displacements, displayed as the approximated percentage of trajectories that finished within each 45° sector of the experimental chamber, represented here as a polar chart. A′ (right panel), B′–D′, E′ (right panel) and F′–H′) displacement cosines corresponding to panels (A–D) and (E–H). The experimental duration was 34′10″ and 50 amoebas were used per scenario. “p” chemotactic peptide (nFMLP) location; “+” anode location; “−” cathode location. The starting point for each trajectory is located at the center of the plot, and the distance in millimeter is shown on both the *x-* and *y*-axes.

The locomotion trajectories of 50 cells and 50 cytoplasts in a stimulus-free medium were monitored for 34 min and 10 s (Fig. 3A and E). Movement directionality was quantified by computing the angle of displacement (Fig. 3A′ and E′, left panels) and the displacement cosine for each trajectory (Fig. 3A′ and E′, right panels). Cosine values close to −1 imply a leftward bias, while values close to 1 indicate a rightward bias. Our analysis revealed that values spanned between −1 and 1. Median/IQR values for each cell type were −0.28/1.28 (cells) and 0.09/1.40 (cytoplasts), with an overall median/IQR of −0.05/1. These results suggest that amoebae moved with no preferred direction in the absence of stimuli. BEID: *N* = 50, Er = 17, Nr = 1–4 (cells); *N* = 50, Er = 34, Nr = 1–4 (cytoplasts).

### Effects of an electric field on cell displacement

We recorded the trajectories of 50 cells and 50 cytoplasts migrating in the presence of a 300–600 mV/mm electric field (Fig. 3B and F). Amoebae overwhelmingly migrated toward the cathode, located on the right side of the setup. The displacement angle cosines for all trajectories were calculated to assess movement directionality. Median/IQR values were 0.98/0.13 for cells, 0.96/0.12 for cytoplasts, and 0.97/0.13 for all trajectories (Fig. 3B′ and F′), confirming the emergence of a new behavior in the presence of an electric field. The results of a Wilcoxon rank-sum test comparing values for the displacement cosines in the absence of stimuli and under an electric field showed that a new migration pattern with an extremely low chance to occur at random emerged in both cells and cytoplasts. (*P*-values: 10^−12^ and 10^−9^; *Z*: −7.08 and −5.98 for cells and cytoplasts, respectively). BEID: *N* = 50, Er = 11, Nr = 4–8 (cells); *N* = 50, Er = 29, Nr = 1–4 (cytoplasts).

### Effects of a peptide chemical gradient on cell locomotion

We observed the migration trajectories of 50 cells and 50 cytoplasts under a chemotactic gradient of nFMLP peptide, which was applied on the left side of the experimental setup (Fig. 3C and G). Most of the amoebae (83%) moved toward the source of the peptide. We calculated the displacement angle cosines for each of the 100 trajectories, which ranged from −1 to 1. The median/IQR values were −0.66/0.65 for cells, −0.71/0.57 for cytoplasts, and −0.70/0.65 for all trajectories, suggesting a consistent pattern of movement toward the peptide (Fig. 3C′ and G′). We used the Wilcoxon rank-sum test to compare the cosine values in the presence of an electric field or a chemotactic gradient (*P*-values: 10^−13^ and 10^−15^; *Z*: 7.43 and 7.88 for cells and cytoplasts, respectively). These results indicated that the chemotactic gradient induced a distinct locomotion behavior from the one elicited by the presence of an electric field both in cells and in cytoplasts. BEID: *N* = 50, Er = 12, Nr = 3–7 (cells); *N* = 50, Er = 21, Nr = 1–4 (cytoplasts).

### Cell migration under combined electric and chemical stimuli

We analyzed the migratory trajectories of 100 amoebae (50 cells and 50 cytoplasts) under the simultaneous application of an electric field and a chemotactic gradient of nFMLP peptide. The peptide was located on the left side of the setup (anode), while the right side was the cathode (Fig. 3D and H). In this condition, we observed that 42% of the amoebae moved toward the cathode, while 58% moved toward the peptide (anode). The median/IQR values of the displacement angle cosines were −0.40/1.76 for cells, −0.55/1.41 for cytoplasts, and −0.44/1.60 for the 100 trajectories. These values indicated that the amoebae exhibited two distinct migratory behaviors, one toward the anode and one toward the cathode (Fig. 3D′ and H′). The Wilcoxon rank-sum test revealed that these behaviors were significantly different for both cells (*P* = 10^−9^; *Z* = 5.98) and cytoplasts (*P* = 10^−9^; *Z* = 5.98). BEID: *N* = 50, Er = 13, Nr = 3–4 (cells); *N* = 50, Er = 15, Nr = 2–4 (cytoplasts).

### Influence of long-range correlations on cellular locomotion

Complex systems exhibit systemic behavior that is characterized by the existence of long-range correlated dynamics (64). A widely used technique to detect these correlations in time series data (such as migratory patterns in this case) is the “root mean square fluctuation” (“rmsf”) analysis, a classic approach in Statistical Mechanics that draws on the concepts proposed by Gibbs (65) and Einstein (66).

**Fig. 4.**
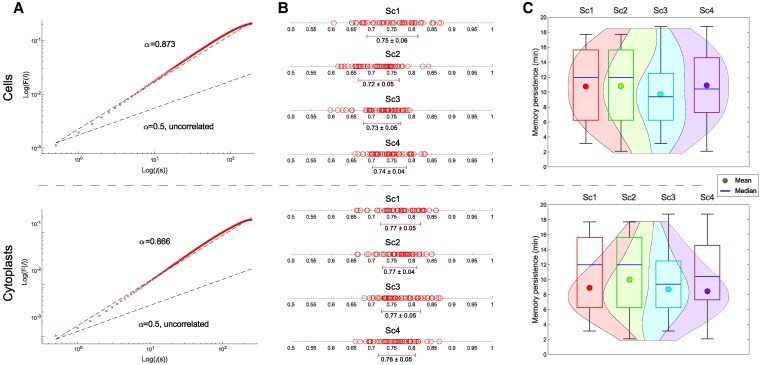
Move-steps of cellular migratory displacements show mutual interdependence over long periods of time. A) Log–log plots of rmsf *F* versus *l* step for a prototypical *A. proteus* cell belonging to scenario 1 (Sc1, upper panel) and cytoplast belonging to scenario 3 (Sc3, lower panel). B) Linear graphs display the experimental rmsf correlation coefficient *α* values (and global average ± SD) for all cells (upper panel) and cytoplasts (lower panel) under each experimental scenario. C) The violin plots depict the distribution, mean, and median of correlation duration values for cells (upper panel) and cytoplasts (lower panel) in the four experimental scenarios. “Sc1” scenario 1 (no stimuli); “Sc2” scenario 2 (galvanotaxis under an electric field); “Sc3” scenario 3 (chemotaxis along a peptide gradient); and “Sc4” scenario 4 (simultaneous galvanotactic and chemotactic cues).

A power-law relation of the form $F(l)∼l^{\alpha }$ can reveal long-range interdependence, where *l* is the number of steps. The fluctuation exponent *α* is around 0.5 for uncorrelated data, while α>0.5 or α<0.5 signify the existence of positive or negative long-range correlations, respectively (see SI Appendix, Supplementary Methods). Figure 4A shows an example of “rmsf” analysis for the locomotion patterns of a typical cell (taken from scenario 1) and cytoplast (taken from scenario 3).

To test the reliability of this technique, we shuffled all the 400 experimental trajectories (2 × 10^5^ shuffles per trajectory) and compared their scaling exponent α values with the original ones. Figure 4B displays the “rmsf” analysis results of the 400 experimental amoeba trajectories. The cellular move-step migratory fluctuations of all the experimental trajectories showed nontrivial correlations. We observed that the “rmsf” scaling exponent *α* had a median/IQR of 0.74/0.07 for cells and 0.77/0.07 for cytoplasts, with values for the experimental trajectories ranging from 0.60 to 0.87, with a median/IQR of 0.75/0.07, and values for all shuffled trajectories ranging from 0.37 to 0.62, with a median/IQR of 0.47/0.07 (see SI Appendix, Tables S1 and S2 for more details). A Wilcoxon rank-sum test revealed highly significant differences in *α* values between experimental and shuffled trajectories (*P* ≅ 0, *Z* = 24.48), suggesting the unlikelihood of random occurrence of the observed long-range correlations in the experimental data.

We also measured the time span of the correlations regime and observed that both cells and cytoplasts displayed long-range correlations with a median/IQR value of 10.42/8.33 and 8.33/5.21 min, respectively, which emphasizes the effect of prior trajectory values on each move-step. All amoebae showed long-range coordination over periods varying from 2.08 to 18.75 min with a median/IQR of 9.38/7.29 min. These results imply strong dependencies of previous movements (Fig. 4C and SI Appendix, Table S3). These findings indicate the existence of nontrivial long-range correlations in all displacement trajectories.

### Efficient system dynamics in cellular directed movement

The strong anomalous dynamic of cell migration is another feature of cell locomotion. This is linked to anomalous super-diffusion, a complex process that is nonlinear with respect to time and leads to effective directional trajectories at the systemic level (67, 68). We calculated the mean square displacement (MSD), a method developed by Einstein (69) and von Smoluchowski (70), to measure this dynamic property. This tool from Statistical Mechanics enables the quantification of the space covered by the amoebae during their movement. The anomalous diffusion exponent *β* is used in this method to indicate if normal (Brownian, β≅1) or anomalous diffusion (β⧸≅1) occurs (see SI Appendix, Supplementary Methods). Subdiffusion and superdiffusion dynamics are associated with 0<β<1 and β>1, respectively.

**Fig. 5.**
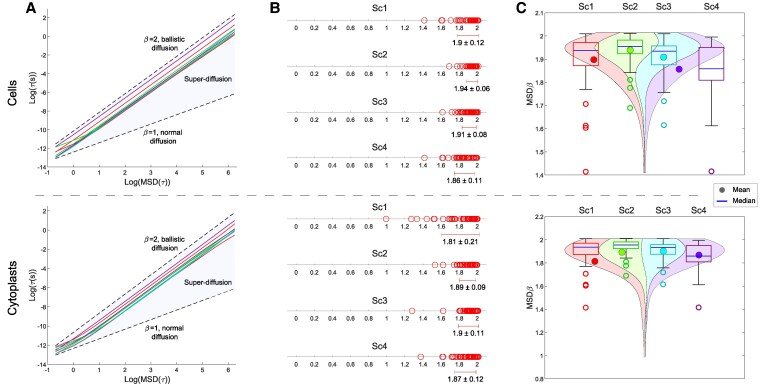
Strong anomalous migration patterns in cellular movement. A) *β* values of the MSD for eight representative cells (upper panel) and cytoplasts (lower panel) belonging to scenario 1 (Sc1). β=1 indicates normal diffusion, β=2 ballistic diffusion, and the shaded region that lies in between is indicative of superdiffusion, a complex process exhibiting a pronounced nonlinear correlation with time, encompassing all the observed values of the *β* exponent from the experimental trajectories. B) The linear graphs illustrate all *β* exponent values (and their global average ± SD) for cells (upper panel) and cytoplasts (lower panel) under the four experimental scenarios. C) The violin plots show the estimated distribution, median, and mean of the MSD *β* exponent values from all experimental cell (upper panel) and cytoplast (lower panel) trajectories. “Sc1” scenario 1 (no stimuli); “Sc2” scenario 2 (galvanotaxis under an electric field); “Sc3” scenario 3 (chemotaxis along a peptide gradient); and “Sc4” scenario 4 (simultaneous galvanotactic and chemotactic cues).

We performed an MSD analysis of the locomotion movements of eight representative cells and cytoplasts without stimuli, shown in Fig. 5A. The MSD analysis of the 400 experimental cells (Fig. 5B and C and SI Appendix, Table S4) revealed that almost all trajectories display strong anomalous migratory dynamics. The variable *β*, which describes the diffusion process, had a median/IQR value of 1.93/0.11 for cells and 1.91/0.11 for cytoplasts in the experimental trajectories. These values indicate an anomalous superdiffusive process, a complex behavior that seems to dominate the cell trajectories in both groups.

Values of the anomalous diffusion exponent *β* for all experimental trajectories ranged from 0.99 to 2.01, with a median/IQR value of 1.92/0.12, suggesting a superdiffusive behavior, while the shuffled trajectories had values ranging from −0.00 to 0.01, with a median/IQR value of 10^−4^/0.00, extremely subdiffusive values that imply highly ineffective exploration patterns (Fig. 5B and SI Appendix, Table S5). A Wilcoxon test comparing experimental with shuffled anomalous diffusion exponents demonstrated that our results are highly improbable to occur by chance (*P* ≅ 0, *Z* = 24.48).

### Unpredictability and information content in migratory trajectories

We used the approximate entropy (ApEn), a reliable estimate of the Kolmogorov–Sinai (K-S) entropy (71, 72), to measure the information content within locomotion trajectories and shed light on the complex migratory behavior that results from the cellular system. ApEn, introduced by Pincus et al. (73), serves as a statistical tool to quantify the regularity and complexity of fluctuations in time series data. A series with intricate and challenging-to-anticipate behavior demonstrates high ApEn, while a series containing repetitive patterns shows low ApEn.

**Fig. 6.**
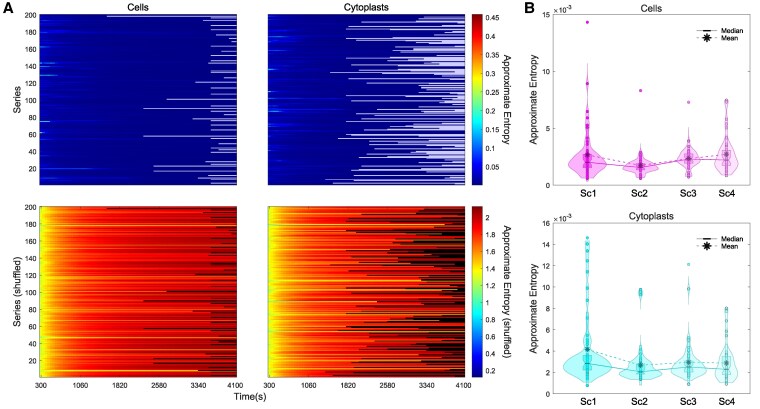
Unpredictability in cellular migration. A) Heatmaps of ApEn values for all 400 experimental (upper row panels) and shuffled (lower row panels) cell (left panels) and cytoplast (right panels) trajectories. ApEn is a statistical tool used to determine the consistency and foreseeability of a time series. A signal with recurring patterns will have a low ApEn, indicating regularity, whereas a high ApEn is associated with an irregular and less predictable series. Every row in the heatmaps corresponds to one individual trajectory and the 82 columns display the number of data points used for ApEn calculation, increased in 50 data points (25 s) at every iteration. B) The violin plots display the estimated distribution, mean, and median of the ApEn values for all the experimental cell and cytoplast trajectories. “Sc1” scenario 1 (no stimuli); “Sc2” scenario 2 (galvanotaxis under an electric field); “Sc3” scenario 3 (chemotaxis along a peptide gradient); and “Sc4” scenario 4 (simultaneous galvanotactic and chemotactic cues).

The results of ApEn estimation for the 400 experimental and shuffled trajectories are shown in Fig. 6 (SI Appendix, Tables S6 and S7, respectively). In Fig. 6A, the ApEn values corresponding to intervals with fewer than 300 data points were omitted from the heatmaps. This exclusion is due to ApEn requiring a minimum of 10*^m^* data points (where *m* = 2 in our calculations) to yield meaningful results (73). Notably, such short time series with a length of ≤200 points are considered unreliable for ApEn analysis (74). The heatmaps illustrate the Approximate K-S entropy values for the experimental (top row) and shuffled trajectories (bottom row) from cells (left panels) and cytoplasts (right panels), computed for 82 different time windows (intervals) with increasing length (we increased the interval duration by 25 s at each iteration). The intervals have ApEn values ranging from 10^−4^ (blue) to 0.46 (red) for experimental trajectories and from 0.12 (blue) to 2.13 (red) for shuffled trajectories. These results reveal that almost all the experimental series have very low entropy. To enhance visualization, we included in the ranges of the heatmap color maps of Fig. 6A the interval ApEn values corresponding to the 300 first data points.

The ApEn analysis of experimental trajectories yielded a narrow spectrum of very low values ranging from 10^−4^ to 0.02, a median/IQR of 0.002/0.001 for cells, 0.003/0.001 for cytoplasts, and an overall median/IQR of 0.002/0.001.

Conversely, the ApEn for shuffled trajectories had a range of very high values (from 1.30 to 2.13, median/IQR of 1.96/0.20) compared with the values for experimental trajectories (SI Appendix, Table S7). Our analysis verifies the existence of a complex structure with high information content in the move-step sequences of the cell migration trajectories. Moreover, the results of a Wilcoxon rank-sum test comparing the respective ApEn value distributions of the experimental and shuffled trajectories (*P* ≅ 0 and *Z* = −24.48) evidence that the complex dynamic structure seen in the move-step series was extremely improbable to be randomly obtained.

### Persistence in cellular migration

Another key feature of the systemic cellular migration movements in unicellular organisms is the long-range memory effects or persistence (75, 76). The detrended fluctuation analysis (DFA) (see SI Appendix, Supplementary Methods) is a widely used method for detecting persistent effects in physiological time series.

**Fig. 7.**
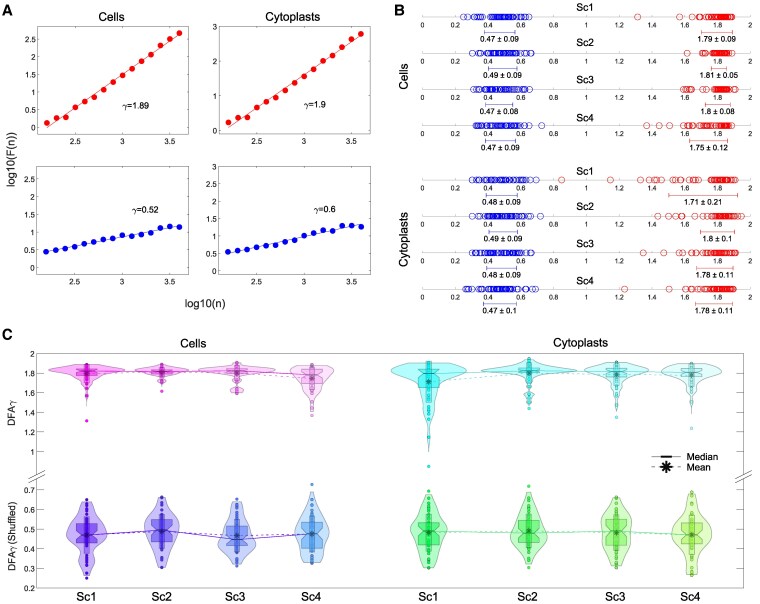
Long-term memory influences the migratory behavior of cells. A) The detrended fluctuation parameter F(n) and the window size *n* for both a typical cell (left panels) and a cytoplast (right panels) in the absence of stimuli (Sc1) are displayed on a log–log chart. The large scaling exponent *γ* values indicate long-range memory effects in both cells and cytoplasts. By shuffling, the original trajectories lost their memory information, which made the *γ* values decrease from *γ* = 1.89 to *γ* = 0.52 in the cell and from *γ* = 1.9 to *γ* = 0.6 in the cytoplast. B) The scaling exponent *γ* value (and its average ± SD) for all cells and cytoplasts in every experimental scenario (Sc1–Sc4) is shown, experimental values in red and shuffled values in blue. C) The violin plots depict the approximate distribution of scaling exponent *γ* values, along with their mean and median, for all experimental (top panel) and shuffled (lower panel) cell (left) and cytoplast (right) trajectories. “Sc1” scenario 1 (no stimuli); “Sc2” scenario 2 (galvanotaxis under an electric field); “Sc3” scenario 3 (chemotaxis along a peptide gradient); and “Sc4” scenario 4 (simultaneous galvanotactic and chemotactic cues).

For a specified observation scale, denoted as *l*, DFA computes the function F(l) to quantify the fluctuations of the time series around its local trend. If the time series exhibits scaling properties, then $F(l)∼l^{\left(\gamma \right)}$ asymptotically, where *γ* is the scaling exponent. This exponent is typically estimated as the slope of a linear fit in the log-log plot of F(l) versus *l*. Therefore, *γ* indicates the degree of long-term memory effects and describes the underlying dynamical system. Note that motion in the experimental time series was Brownian, becoming Gaussian white noise after shuffling. For Gaussian white noise, 0<γ<0.5 means antipersistent correlation, values near 0.5 imply the lack of long-range correlations, and 0.5<γ<1 indicates persistence. In contrast, a Brownian process shows antipersistence when 1<γ<1.5, no correlation when γ≈1.5, and positive long-range persistence when 1.5<γ<2 (77) (Fig. 7A). By applying this quantitative method, we detected the existence of long-range persistence in all experimental trajectories (SI Appendix, Table S8). In particular, the median/IQR DFA scaling parameter *γ* was determined to be 1.82/0.08 for cells and 1.82/0.09 for cytoplasts, with an overall value of 1.82/0.08 (Fig. 7B and SI Appendix, Table S8), thereby indicating that all the move-step series display trend-strengthening behavior.

The strong correlation values observed in the experimental migration series disappeared after shuffling (see Fig. 7B and C and SI Appendix, Table S9 for more details), with an overall median/IQR γ value of 0.48/0.12. This finding verifies that the complex locomotion structure, characterized by well-structured move-step sequences and persistent dynamics observed in the migration trajectories of the two cell groups, is not due to a random chance (*P*  ≅ 0, *Z* = 24.48).

### Kinematic properties of cellular motion

**Fig. 8.**
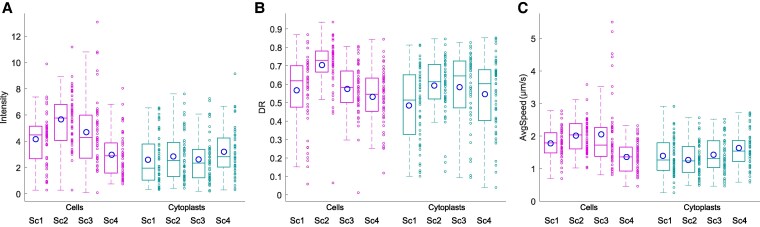
Kinematic characteristics of cell migration. A–C) The group boxplots represent the distributions of all experimental values for three key cytokinematic metrics, which unveil fundamental information about the traits of cell migration—(A) Intensity of the response, (B) DR, and (C) AS (in µm/s)—for the two cell types (enucleated and nonenucleated) under all four experimental scenarios (Sc1, Sc2, Sc3, and Sc4).

We measured the intensity of the response (IR), the directionality ratio (DR), and the average speed (AS) of amoebae (Fig. 8A–C) to evaluate some kinematic aspects of the cell migration trajectories.

The IR reflects the space covered by the cell, and specifically, we computed the modulus of the trajectories to indicate the magnitude of the response. The median/IQR IR was 4.31/3.04 for cells and 2.47/2.42 for cytoplasts. Then, we examined the DR, which calculates trajectory linearity values ranging between 0 (for completely curved trajectories) and 1 (for completely straight trajectories) by using the initial and final positions of the path taken by the amoeba. Experimental values ranged from 0.01 to 0.94 (median/IQR 0.62/0.21) for cells and 0.04 to 0.85 (median/IQR 0.60/0.28) for cytoplasts.

Lastly, we estimated the AS of the trajectories, which varied between 0.46 and 5.50 µm/s (median/IQR 1.70/0.78) for cells and 0.26 and 2.92 µm/s (median/IQR 1.34/0.84) for cytoplasts.

### Systemic properties of cell displacement

**Fig. 9.**
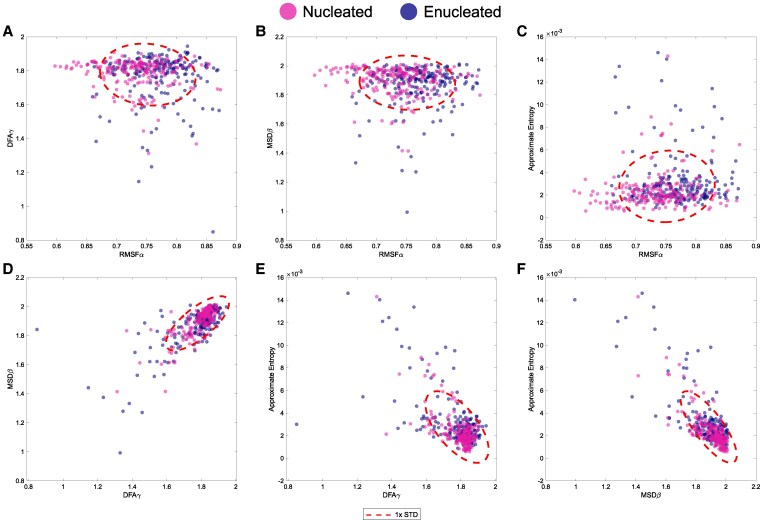
Systemic properties of cell migration. A–F) The results of the analysis of the 400 experimental trajectories, with key metrics considered in our work, namely RMSF Alpha, DFA Gamma, MSD Beta, and ApEn. The values for cells (in pink) and cytoplasts (in blue) overlap in all comparisons, being qualitatively very similar. Ellipses representing one times the standard deviation of the data in each data “cloud” have been drawn to facilitate data interpretation.

In Fig. 9A–F, we performed several pairwise visual comparisons of the main metrics considered in our experimental study (RMSF Alpha, DFA Gamma, MSD Beta, and ApEn). All *P*-values of pairwise and group comparisons between experimental scenarios and cell types for each metric are shown in SI Appendix, Table S10. The values for cells and cytoplasts overlap in all comparisons, showing high similarity. Red ellipses of size one times the SD of each cluster have been overlaid on the data for better visualization. Data from nonenucleated and enucleated cells were clustered together in every comparison for the experimental time series, indicative of their high similarity (see Fig. 9A–F).

The results of all the quantitative analyses performed were qualitatively similar for both nonenucleated and enucleated cells (Table 1).

Finally, we have performed different types of alternative control experiments to complete the results obtained (see SI Appendix, Figs. S2–S8 and Tables S11–S15).

## Discussion

Cellular migratory displacements represent an extremely complex tightly regulated process, essential in all Metazoan organisms. Deregulated locomotion movements are involved in important human diseases such as immunodeficiencies and cancer.

Despite its importance in a plethora of critical cellular functions, how migratory cells coordinate the intracellular physiological processes, the subcellular organelles, and molecular-metabolic responses is an unresolved issue in contemporary biology.

Recently, in a previous work, we have shown that the cellular movements are regulated by integrative self-organized molecular processes operating at the systemic level (33).

Here, to continue the study of the systemic dynamic forces driving the migratory displacements, we have tested whether this type of emergent systemic behavior also occurs under extreme physiological conditions such as those that operate in enucleated cells. Toward this goal, we have quantitatively analyzed the movement trajectories of 200 individual enucleated cells (cytoplasts) and 200 nonenucleated cells belonging to *A. proteus* species (400 cells in total). All the locomotion trajectories have been obtained in four different experimental scenarios: in the absence of stimuli, under a galvanotactic field, in a chemotactic gradient, and under complex conditions such as simultaneous galvanotactic and chemotactic stimuli.

First, we have analyzed the long-range interdependence in the move-step sequences of the cell displacements using the “rmsf” method rooted in Statistical Mechanics, drawing upon foundational concepts developed by Gibbs and Einstein (65, 66, 78–80). The “rmsf” obtained values have been similar for both nonenucleated cells and cytoplasts, and specifically, indicated robust dependencies over periods of 9.38/7.29 min (10.42/8.33 min for nonenucleated cells and 8.33/5.21 for cytoplasts, median/IQR). The enucleated and nonenucleated unicellular organisms exhibited nontrivial long-range correlations in all their directional trajectories, a hallmark of an emergent systemic dynamics within the cell (76).

Furthermore, we identified anomalous behavior in migratory movements using the MSD method, also proposed by Einstein (69). Particularly, we have observed that both nonenucleated and enucleated amoebas show similar values of superdiffusion (1.93/0.11 for cells and 1.91/0.11 for cytoplasts, median/IQR value). Likewise, the MSD is a proxy for the surface area explored by each cell over time, and is a measure related to the overall migration efficiency, which corresponds to adequate movements during exploration of the extracellular medium (67, 68, 81).

We have also quantified the regularity and unpredictability of fluctuations during migratory displacements using ApEn (71, 72). The results revealed substantial information in all analyzed trajectories. The entropy was remarkably low in all the directional movements, suggesting that the migration patterns operate at a higher complexity level. These values confirm the existence of a highly complex structure of move-step sequences in the cellular displacements. The results also showed that such dynamic complexity in trajectories is highly improbable to occur by chance.

In addition, we have verified the presence of “long-term memory effects” in all cellular migratory movements of both types of unicellular organisms. Specifically, the DFA fluctuation analysis (82) indicated that move-step trajectories exhibit trend-reinforcing memory, i.e. past directional movements showing increased move-step values are likely to be followed by future increasing trends, and vice versa. Therefore, DFA results also validate the presence of strong long-range correlations in locomotion movements observed by the “rmsf” analysis.

Finally, we have analyzed three main kinematic properties of cellular motion, obtaining similar DR and AS values for both nonenucleated and enucleated cells, and finding slight differences in IR.

Specifically, the values obtained from the analysis of “RMSF *α*,” “MSD *β*,” “DFA *γ*,” “ApEn,” “DR,” and “AS” for both the nonenucleated and enucleated amoebas displayed very similar values (see Table 1). However, the “RMSF correlation times” and “IR” showed slightly different values. Note that enucleated cells lack the nucleus, a structure with important mechanic and sensory functions in cell migration that could affect the intensity of the cellular response. In addition, enucleation is a method that damages the plasma membrane, and during which a small part of the cytoplasm is extracted together with the nucleus (see Fig. 2). Although *A. proteus* are very robust organisms, the enucleation might lead to some quantitative differences between the migratory characteristics of nucleated and enucleated cells; particularly we have observed in our experiments that enucleation clearly affects the RMSF correlation times and the intensity of the migratory response of cytoplasts.

We have quantitatively detected that enucleation clearly affects the correlation times and the intensity of the migratory responses of cytoplasts: we found that the average correlation time of the migratory responses (the time during which amoebal movement is influenced by previous steps) was shorter in cytoplasts (8.33/5.21 min) than in intact cells (10.42/8.33 min). The intensity of the migratory response, calculated as the module of the vector connecting the initial and final coordinates of each amoeba trajectory (displacement vector) was also smaller on average in enucleated cells (2.47/2.42 mm) than in intact cells (4.31/3.04 mm) (median/IQR values, see Table 1).

The quantitative studies unequivocally show that in both the nonenucleated and the enucleated amoebas emerges a very complex dynamic structure in the migratory movements of all analyzed cells. Such a structure is characterized by highly organized move-step sequences with a very low level of entropy and high information, nontrivial long-range interdependence in the move-steps, strong anomalous super-diffusion dynamics, long-term memory effects with trend-reinforcing behavior, and efficient movements to explore the extracellular medium.

This type of dynamic structure in the migratory movements is a consequence of the dissipative self-organized dynamics, intrinsic to unicellular organisms (33). Cells are open systems that operate far from the thermodynamic equilibrium and exchange energy-matter with the environment (83–85). Under these conditions, the cellular physiology is characterized by exhibiting a great number of irreversible enzymatic processes in the cellular metabolic networks allowing the system to become spatially and temporally self-organized (86–92). There are different types of dissipative self-ordered biochemical structures in the cell, the most studied are temporal molecular oscillations, molecular circadian rhythms (with oscillatory periods close to 24 h), and spatial traveling waves that correspond to a coherent oscillation of metabolite concentrations that propagates progressively across the intracellular medium (83, 85, 86, 92). Examples of ultradian oscillations involved in the regulation of cell migration include rhythms in the concentration of cytoskeletal actin (93), myosin dynamics (94, 95), rhythmic cAMP levels (96), and oscillations in intracellular Ca^2+^ (97, 98). Temporal oscillations with a period close to 24 h have also been associated with cell migration; for instance, circadian rhythms shaping the concentration of adhesion molecules and of the chemokine CCL21 (99), oscillations in intracellular pericentrin protein levels (100), and daily rhythms in the concentration of actin cytoskeleton regulatory molecules (101). Spatiotemporal waves include intracellular Ca^2+^ waves (97, 102), actin waves (103–105), PIP3 waves (104), NAD(P)H waves (106, 107), and kinase waves (108, 109).

Furthermore, an important question in our work is the role of the nucleus in the migratory movements of the cells. Different biological studies have addressed the mechanical implications of the nucleus in locomotion movements. The physical properties of the nucleus, strongly connected with the cytoskeleton, play an important mechanical function by favoring cellular locomotion (43, 110–114). In fact, we have observed in our experiments that enucleation clearly affects the correlation times and the intensity of the migratory response of cytoplasts, as we described above.

Recently, the nucleus was removed from fibroblasts and endothelial cells in a large study (115), and it was observed that such cytoplasts without nuclei, correctly polarize and migrate on flat 2D surfaces in a similar way to nonnucleated cells, confirming that the nuclear activities are not essential in the locomotion movements of unicellular organisms (116). The authors in (115) additionally determined that the nucleus, through its mechanical properties, particularly its connections with the cytoskeleton, serves as a crucial mechanical component in regulating adequate physical–mechanical responses during cellular migration in 3D collagen matrices. An earlier study had revealed that enucleated fibroblasts can adhere to a 3D cell–derived extracellular matrix and migrate in it, although more slowly than intact cells (117). Another experimental study also showed that nucleated and enucleated leukocytes exhibit comparable cellular functionality and displacements patterns when migrating under various compressive forces applied by agarose patches (118). More recent publications in immunological therapies have revealed that enucleated cells of mesenchymal stroma and melanoma retain their functional properties and migrate in diseased tissues as normal cells; additional studies by the same group have demonstrated analogous cytoskeletal functionality between nucleated and nonnucleated cells (119, 120).

In our extensive quantitative work, with 400 single cells and four different experimental scenarios, we confirmed that both nonenucleated amoebas and cytoplasts display the same complex kind of dynamic migratory structure. The locomotion displacements of enucleated cells seem to be regulated by emergent self-organized molecular dynamics, carefully modulated at a global-systemic level, which seems to depend on the cooperative nonlinear interaction of most of the molecular components of the cytoplasm.

About the role of the nucleus in the migratory movements of the cells, we have observed that enucleation clearly affects two important properties of cell migration, such as the correlation times and the intensity of the migratory response of cytoplasts. However, the fact that cytoplasts preserved the dynamic properties of their locomotion trajectories as well as the nonenucleated cells suggests that the nuclear activity has a minor role in regulating the systemic locomotion displacements of cells.

The obtained quantitative values with enucleated cells corroborate our previous published results (33), which showed that migratory responses in nonenucleated cells seem to be regulated by functional integrative processes operating at the systemic level. This result does not invalidate the notable scientific importance of all studies performed on the influence of individual and particular molecular mechanisms on cell migration.

Cell migration is a central issue in many human physiological and pathological processes, and we consider that new researches combining migratory systemic dynamics with molecular studies may be fundamental for the development of next-generation, efficient cellular therapies for migration disorders.

## Materials and methods

### Cell cultures

*Amoeba proteus* (Carolina Biological Supply, #131306) were cultured at 21 °C in Ø100 × 20 mm Petri dishes (Corning CLS430167) containing Chalkley's simplified medium (63) (NaCl, 1.4 mM; KCl, 0.026 mM; CaCl2, 0.01 mM) and one heat-treated wheat grain.

### Experimental setup arrangement

A schematic representation of the experimental arrangement is presented in Fig. 1. The setup involves two electrophoresis blocks (Bio-Rad Mini-Sub cell GT) put together in such a way that the first electrophoresis block is connected to a BIO-RAD Model 1000/500 power supply unit and to the second block by two 12-cm-long 2% agar bridges containing a 0.5 N KCl solution. Notably, the use of agar bridges and the removal of all electrodes from the second electrophoresis block serve to prevent direct contact between the moving cells and any metallic electrodes, thereby mitigating the risk of ionic contamination.

All experimental replicates were conducted using small groups of size 2.63 ± 1.42 (mean ± SD) and a maximum of eight cells and had a duration of 34 min and 10 s. Throughout this duration, cell behavior was meticulously recorded using a digital camera. Prior to each experiment, amoebae were subjected to a 24-h starvation period in fresh, nonnutritive simplified Chalkley's medium. Only cells exhibiting optimal vitality (characterized by motility and spindle-shaped morphology) were selected for the experiments. Notably, deviations from optimal culture and experimental conditions, as well as mechanical anomalies within the recording system, led to the exclusion of ∼10% of the experimental replicates. These invalidated replicates were not considered for subsequent analysis.

The experiments were conducted within a custom-devised experimental glass chamber, put over the central elevated platform of the second electrophoresis block, where the amoebae were placed and could freely crawl. It consisted of a modified standard glass slide of dimensions 25 × 75 × 1 mm to which two 24 × 60 × 0.17 mm cover glasses were glued with silicone adhesive. After allowing the assembly to dry for 24 h, the overhanging portions (measuring 20 × 60 × 0.17 mm) from both cover glasses were cut off, leaving two 4 × 60 × 0.17 mm glass sheets attached to the glass slide that served as the chamber's sidewalls. Finally, three additional smaller glass pieces, crafted by carefully trimming three 24 × 60 × 0.17 mm cover glasses, were placed over the modified glass slide: a central piece measuring 3 × 24 × 0.17 mm and two larger pieces flanking it, each measuring 24 × 40 × 0.17 mm. The two flanking pieces could be slid toward or away from the central piece to close or open the experimental glass chamber to respectively facilitate the establishment of a laminar flow or allow for the placement and extraction of cells.

New agar bridges were employed in each experimental replicate to prevent contamination of either the agar or the medium and minimize conductivity loss, caused by the leak of KCl from the agar brides into the simplified Chalkley's medium (which possesses a significantly lower osmotic concentration than the agar bridges). Additionally, a dedicated electrophoresis block was exclusively allocated for experiments involving chemotaxis, while another block served specifically for galvanotaxis. Furthermore, the entire experimental setup, including both the electrophoresis blocks and the experimental glass chamber, underwent meticulous cleaning and reassembly after each experimental replicate.

Prior to each experimental replicate, the modified glass slide was affixed to the upper surface of the central platform of the second electrophoresis block. This attachment was achieved using a droplet of olive oil, applied to prevent both the medium and the electric current from circulating beneath the experimental glass chamber. Subsequently, the central glass piece, measuring 3 × 24 × 0.17 mm, was gently positioned atop the center of the modified glass slide. Following this, amoebae were carefully washed twice in fresh simplified Chalkley's medium, placed beneath the central glass piece within the glass chamber and let to attach to the surface of the modified glass slide until they showed an elongated shape and were firmly attached to the substrate, which took an average of ∼2 min. During this step, it is crucial to complete the process within 15 s to prevent the amoebae from adhering to the inner surface of the plastic micropipette tip, which could potentially damage their cellular membranes. To complete the assembly, the two lateral sliding glass pieces, each measuring 24 × 40 × 0.17 mm, were positioned flanking the central piece, slightly overhanging the wells of the electrophoresis block. Subsequently, each well was meticulously filled with 75 mL of clean simplified Chalkley's medium. The lateral glass pieces were gently pressed down using a micropipette tip until they made contact with the medium, which spread beneath them due to surface tension. Finally, the two lateral glass pieces were longitudinally slid until they touched the central piece where the amoebae were situated underneath. This action effectively closed the glass chamber, establishing a connection between the media in both wells.

### Enucleation

Amoebae underwent a double washing in Simplified Chalkley's medium and were subsequently enucleated on 35 × 10 mm Petri dishes using a Sutter Instruments Co. MP-285 micromanipulator and an Olympus CK40 microscope. The enucleation procedure involved delicately introducing a slender glass pipette into the amoeba's cytoplasm through the cell membrane, followed by manual extraction of the nucleus (see Fig. 2C and D for more information). It is important to acknowledge that the enucleation process is notably forceful, resulting in significant damage to the plasma membrane and the removal of a portion of the cytoplasm along with the nucleus. Consequently, this technique was limited to a maximum of two attempts per cell. Once successfully enucleated, the cytoplasts remained undisturbed until they exhibited an elongated shape with few or no thin pseudopodia and were firmly attached to the substrate, which occurred on average ∼2 min after enucleation and were then placed in the setup.

### Fluorescent staining to confirm enucleation

To confirm nucleus removal and verify that the cell membrane was intact, enucleated cells were stained with DAPI after each experiment and photographed in a fluorescence microscopy together with an also DAPI-stained control cell with its nucleus intact. Amoebae were first fixated in 4% paraformaldehyde (ENMA Bio Ltd, #28794.295) for 5 min. Following fixation, they were simultaneously permeabilized with Triton-X 100 (0.1%) and stained with DAPI (1 µg/mL) for 10 min. Finally, observation was carried out using an Olympus IX71 inverted fluorescence microscope at the High Resolution and Analytic Microscopy Service (SGIker) of the University of the Basque Country (UPV/EHU). Of all the enucleations performed, on six occasions out of 296 (which represents 2.03% of all the enucleations performed), we have observed that after enucleation and experimentation, the DAPI staining had a positive outcome, revealing the presence of a nucleus.

### Galvanotactic stimulus

A stable 60 V direct current was applied throughout the experimental setup for the duration of the experiment. The galvanotactic stimulus can have traumatic effects on amoebal behavior. To mitigate these effects, we implemented three key steps in our experiments involving galvanotaxis (see Fig. 1A for more detail):

1. Stable power supply: We programmed the power supply unit to deliver a consistent 60 V direct current.
2. Chamber customization: By adjusting the cross-sectional area of the experimental glass chamber through changing the amount of silicone adhesive used, we optimized the height of the longitudinal walls of the modified glass slide and thus the baseline resistance of the setup.
3. Real-time resistance management: We installed a variable 1 MΩ resistor and an ammeter in series (in that order). Real-time adjustments were made by manually turning the variable resistor's screw to fine-tune the overall resistance of the setup. This ensured a stable 60 V electric potential and optimal current intensity values of 70–74 µA (33) throughout the galvanotactic experiments.

Interestingly, certain amoebae populations exhibited anomalous, inverted, or null responses to the galvanotactic stimulus. To exclude them from our study, we conducted a 5-min galvanotactic test using intensity and voltage values within the optimal range before carrying out any experiments where amoebae were exposed to the galvanotactic stimulus for the first time.

### Chemotactic peptide gradient

To establish a chemotactic peptide gradient, we introduced 750 µL of nFMLP (Sigma-Aldrich, #F3506) at a concentration of 2 × 10^−6^ M into one well of the second electrophoresis block. The medium in this well was then carefully stirred to ensure thorough mixing of the peptide until the amoebae exhibited a response.

To evaluate the nFMLP peptide gradient concentration, we conducted an experiment where 60 µL of medium was sampled at specific time intervals (0, 2, 5, 10, 15, 20, and 30 min) following the addition of nFMLP (SI Appendix, Fig. S1). These samples were extracted from the center of the experimental glass chamber through a narrow gap created by slightly shifting the lateral glass piece adjacent to the central glass component. By referencing known fluorescein-tagged peptide concentration values from a standard curve, we extrapolated the nFMLP concentration at each time point. The procedure involved taking two samples at each time interval and repeating the entire process three times, resulting in a total of six measurements for each time point. Fluorescence measurements were performed at 460/528 excitation/emission wavelengths using 96-well glass bottom black plates (Cellvis, #P96-1.5H-N) and a SynergyHTX plate reader (BioTek), following the established protocol by Green and Sambrook (121).

### Trajectory recording and analysis

The motility of each cell was documented using a MU500 AmScope digital camera, which was affixed to the trinocular port of an AmScope SM-2T stereomicroscope. The stereoscope's trinocular port included a built-in 0.5× reduction lens, which in conjunction with a 0.75× reduction Barlow lens attached to the stereoscope's objective allowed us to increase the field of view to ∼3.3 × 2.5 cm. Data were collected at a rate of two images per second over a period of 34 min and 10 s (4,100 frames). The inclusion criterion stipulated that only amoebae that were still actively moving 15 min after being enucleated would be considered. To ensure precision, manual tracking was conducted using the TrackMate (122) software within ImageJ. This approach was preferred over automated tracking software, which is often prone to inaccuracies (123), and to require “post hoc” manual error correction (124). Each tracked trajectory corresponded to a distinct amoeba, and no individual amoeba was recorded more than once. A total of 400 digitized trajectories, evenly divided into two groups (enucleated and nonenucleated), were subjected to analysis.

### Statistical analyses

In our study, we initially evaluated the normality of the distribution of our quantitative data using the Kolmogorov–Smirnov test for single samples. However, due to the rejection of normality, we assessed the significance of our findings using two nonparametric tests: The Kruskal–Wallis test to compare multiple groups and the Wilcoxon rank-sum test for pairwise group comparisons. Since both tests are nonparametric, we reported our results in terms of median and IQR, rather than mean ± SD. Additionally, we included *P*-values and *Z* statistics to provide a comprehensive understanding of the statistical significance.

## Supplementary Material

pgaf232_Supplementary_Data

## Data Availability

The data generated by this study are publicly accessible from the Harvard Dataverse repository at: https://doi.org/10.7910/DVN/SA2S7P. This study did not generate any code. Any additional information required to reanalyze the data reported in this paper is available from the lead contact upon request.

## Preprints

This manuscript was posted on a preprint at https://www.biorxiv.org/content/10.1101/2024.05.19.594852v1.
