# Supplementary material for: Migratory responses in enucleated cells: The forces driving the locomotion movement of unicellular organisms
Source: PNAS Nexus. 2025 Jul 25;4(8):pgaf232. doi: 10.1093/pnasnexus/pgaf232 (PMC12341899; doi:10.1093/pnasnexus/pgaf232)
Supplement: pgaf232_Supplementary_Data [file pgaf232_supplementary_data.pdf]

Supporting Information for

Migratory responses in enucleated cells: the forces driving the locomotion movement of unicellular organisms

Ildefonso M. De la Fuente, Jose Carrasco-Pujante, Maria Fedetz, Carlos Bringas Roldan, Alberto Pérez-Samartín, Gorka Pérez-Yarza, Luis Martínez, José I. López, Jesus M Cortes*, and Iker Malaina*

*** Equal contribution**

Dr. Ildefonso M. De la Fuente
Email: mtpmadei@ehu.eus
ildefonso@cebas.csic.es

This PDF file includes:

Supplementary Methods
Figures S1 to S8
Tables S1 to S15
SI References

Supplementary Methods

Root-mean-square fluctuation (rmsf) analysis. The rmsf (root-mean-square fluctuation) analysis, is a classic Statistical Mechanics tool grounded in Gibbs' 1902 (1) and Einstein's 1909 (2) work. Later, it was adapted to study physiological signals (3, 4). Utilizing rmsf, we analyzed power-law autocorrelation within move-step fluctuation time-series, consistent with Viswanathan et al.'s 1996 methodology (5). For the calculation of the rmsf in a two-dimensional trajectory $P(t) = [x(t), y(t)]$ with equally distant values in time, the displacement time series is first defined as: $u(t) = \sqrt{[y(t+1) - y(t)]^2 + [x(t+1) - x(t)]^2}$, for $t = 1, \ldots, t_{max}$. The cumulative net displacement after l steps is $y(l) \equiv \sum_{i=1}^{l} u(i)$. The rmsf is then $F(l) \equiv \sqrt{< \Delta y(l)^2 > - < \Delta y(l) >^2}$, where $\Delta y(l) \equiv y(l + l_0) - y(l_0)$ and the brackets represent the average over all possible l_0 values. Thus, $F(l)$ is the square root of the difference between the average of the square of $\Delta y(l)$ minus the square of its average. Persistence is indicated by a power-law relationship in the fluctuations $F(l) \sim l^{\alpha}$. A fluctuation exponent $\alpha = 0.5$ suggests uncorrelated behavior, while $0.5 < \alpha < 1$ indicates persistence and $0 < \alpha < 0.5$ signifies anti-persistence (5).

Mean Square Displacement. Einstein's Mean Squared Displacement (MSD), initially applied to Brownian motion (6), is now widely used for cell motility quantification (7, 8). It is calculated by computing the average squared displacement over increasing time scales (8), indicating the area covered by the cell and correlating with migration efficiency (9). In a 2D path $P(t) = [x(t), y(t)]$, the MSD is given by $MSD(\tau) \equiv \frac{1}{t_{max}-\tau} \sum_{t=1}^{t_{max}-\tau} \big(r(t+\tau) - r(t)\big)^2$, where $r(t) = \sqrt{\big(x(t)\big)^2 + \big(y(t)\big)^2}$ is the instantaneous modulus and τ is the time scale. Diffusion was analyzed up to a time scale of one-fourth the dataset size. Notably, random walks exhibit power law scaling $\mathrm{MSD}(\tau) \sim \tau^{\beta}$, where β delineates diffusion behavior. Specifically, $\beta \cong 1$ indicates uncorrelated Brownian motion, $1 < \beta < 2$ signifies super-diffusive, and $0 < \beta < 1$ indicates sub-diffusive behavior, both of which are indicative of anomalous diffusion often seen in complex systems with persistent characteristics.

Detrended Fluctuation Analysis (DFA). Detrended Fluctuation Analysis (DFA), a method devised by Peng et al., is employed to identify power-law autocorrelations in time series (10). This method is widely applied in analyzing physiological signal patterns (11). For a trajectory time series *u*(*t*), the initial step is to compute the signal profile by summing the deviations of $u(k)$ from its mean, yielding $z(t) = \sum_{k=1}^{t} (u(k) - < u >)$. Subsequently, the series $z(t)$ is segmented into equal-sized intervals of length *n*, and the local trend $z_n(t)$ is removed. The fluctuation of the detrended time series is given by $D(n) = \sqrt{\frac{1}{t_{max}} \sum_{t=1}^{t_{max}} [z(t) - z_n(t)]^2}$. Applying this calculation to all interval sizes reveals a correlation between fluctuation D and interval size n, with the presence of a power-law autocorrelation being indicated by a linear trend on a log-log graph $D(n) \sim n^{\gamma}$. Motion in the experimental time series was Brownian, becoming Gaussian white noise after shuffling. For Gaussian white noise, anti-persistence is observed when $0 < \gamma < 0.5$, values near 0.5 imply the absence of correlations, and $0.5 < \gamma < 1$ indicates persistence. Conversely, a Brownian process is anti-persistent when $1 < \gamma < 1.5$, shows no correlation when $\gamma \approx 1.5$, and positive long-range persistence when $1.5 < \gamma < 2$ holds (12).

Approximate Entropy. Approximate Entropy (ApEn), introduced by Pincus in 1991 (13), assesses the regularity and predictability of a time series. Low ApEn indicates repetitive patterns, whereas high ApEn reflects complexity and unpredictability. To compute the statistic:

For a time series: $U = u(1), u(2), \ldots, u(N)$ of *N* equally spaced values, select a filtering level *r*, often *r* = 0.2·*SD* (*SD* is the standard deviation of the signal) and an integer *m*, the statistic that represents the length of compared runs of data, whose optimal value we estimated following the method outlined by L. Cao (14), resulting in *m* = 2. Formulate vectors $x(1), x(2), \ldots, x(N-m+1)$ in $\mathbb{R}^m$, real m-dimensional space, defined by $x(i) = [u(i), \ldots, u(i+m-1)]$, and for each $i = 1, \ldots, N-m+1$, the sequence $x(1), x(2), \ldots, x(N-m+1)$is used to construct

$$C_i^m(r) = \frac{number\ of\ x(j)\ such\ that\ d[x(i),\ x(j)] \leq r}{(N-m+1)}$$

where *d* [*x*(*i*), *x*(*j*)] is defined as $\max_{0 \leq k \leq m-1} \{|u(i+k) - u(j+k)|\}$.
Here, the distance *d* is the maximum distance between the scalar exponents of vectors *x*(*i*) and *x*(*j*).
Then, $\Phi^m(r) = (N-m+1)^{-1} \sum_{i=1}^{N-m+1} \ln\left(C_i^m(r)\right)$ is determined, where ln is the natural logarithm.
Lastly, define Approximate Entropy as $ApEn(U, r, m) = \Phi^m(r) - \Phi^{m-1}(r)$ for constant *m* and *r*.

Differences between computational methods.

The Mean Square Displacement (MSD) and the Root Mean Square Fluctuation (RMSF) might, from the perspective of the metric they describe, appear redundant or even equivalent, as both suggest that the movement exhibits persistence with a memory time of approximately 10 minutes. However, while both methods provide information on the scaling of jump distances in cellular trajectories, the RMSF specifically relates to fluctuation size of jump distances, and MSD does it in relation to the average jump distance distribution.

The RMSF makes use of the sum over all values of u(l), where l represents the fluctuation size. What we scale is F, which captures the fluctuation between the second moment and its difference from the square of the first moment. Thus, with RMSF, we examine how this fluctuation, across different accumulated jumps from 1 to consecutive steps, scales in a power-law manner.

In contrast, the MSD acts as an average of that fluctuation, as it simply calculates how the mean jump size scales across different cumulative jump sizes. Therefore, while both can provide measures of persistence and, through their scaling, can determine memory times, one focuses on the fluctuations of these jumps, while the other focuses on their average. As such, they are related yet distinct measures, and this is why they use different scaling exponents. Therefore, the MSD is a measure of the deviation of the position with respect to a reference position over time. It is the most common measure of the spatial extent of random motion, and can be thought of as measuring the portion of the system "explored" by the random walker.

While DFA quantifies how the statistics of fluctuation in the jumps trajectory scales —similar to what Root Mean Square Fluctuation (RMSF) does but not Mean Square Displacement (MSD)—the specific way the mean fluctuation is calculated is totally different.

RMSF does not account for any type of stationarity or, in a sense, typical scales that may exist within the series, unlike DFA. In DFA, the jumps and fluctuation sizes are calculated relative to a global average of the entire jump distribution. Recognizing that physiological series are typically non-stationary, DFA incorporates a detrending step, where a trend is removed from the series. This correction accounts for stationarity in different time windows. Thus, DFA employs a fundamentally different approach by using a global average and detrending across varying scales, which RMSF does not do.

While both methods characterize the scaling of observed fluctuation sizes, their methodologies diverge significantly. Lastly, Approximate Entropy is a numerical technique for estimating the entropy of a series, which essentially measures the regularity of oscillations. Higher regularity corresponds to lower entropy and, consequently, greater predictability in the series.

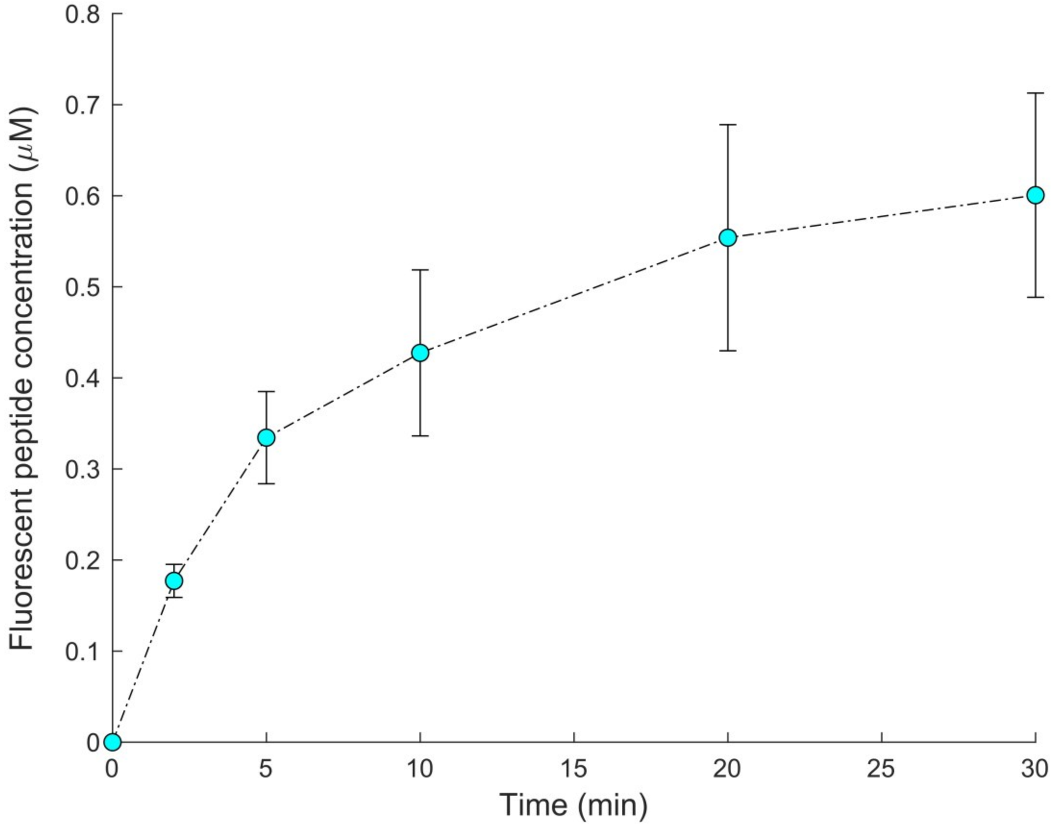

Figure S1. Fluorescein-tagged chemotactic peptide gradient evolution. We measured the temporal dynamics of peptide concentration at the center of the experimental glass chamber. Measurements were taken at specific time intervals (0, 2, 5, 10, 20, and 30 minutes) at the central location where amoebae were positioned. Each data point represents the average concentration (± standard deviation) obtained from six measurements (duplicate sampling in three separate experimental replicates). Notably, the peptide concentration exhibits an initial rise to approximately 0.2 µM within two minutes of establishing laminar flow. Subsequently, it further increases to 0.6 µM by the end of the 30-minute experiment. Adapted from Fig. 2 in https://doi.org/10.1038/s41467-019-11677-w.

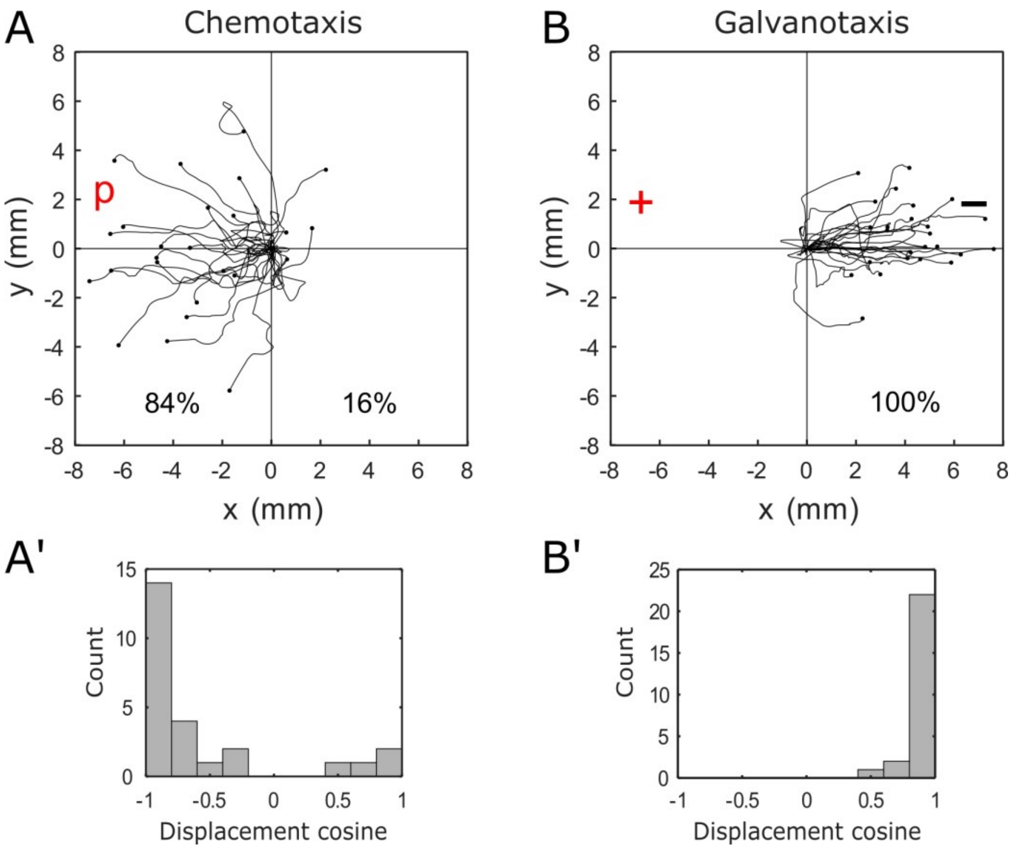

Figure S2. Control tests to check if the exposure to a chemotactic peptide gradient could alter the galvanotactic response of *Amoeba proteus* cytoplasts under an electric field. **(A)** Migration trajectories of 25 *Amoeba proteus* cytoplasts under a chemotactic peptide gradient (nFMLP), where most cells (84%) showed clear directionality towards the peptide. **(B)** The same cytoplasts were then exposed to an electric field. Note that the normal galvanotactic response of all cytoplasts characterized by a directional migration towards the cathode was not altered by the previous exposure to the peptide gradient. **(A', B')** Displacement cosines for the trajectories in (A) and (B). These analyses are consistent with the results obtained in the experiments described in the manuscript, both for cells and cytoplasts, in the chemotaxis and galvanotaxis scenarios. To conclude, the previous exposure to a chemotactic peptide gradient did not alter the cytoplast's normal galvanotactic response under an electric field. Experimental duration was 34'10''. "p" indicates the location of the chemotactic peptide, "+" anode, "–" cathode. BEID: N=25, Er=11, Nr=1-4 (Chemotaxis and Galvanotaxis).

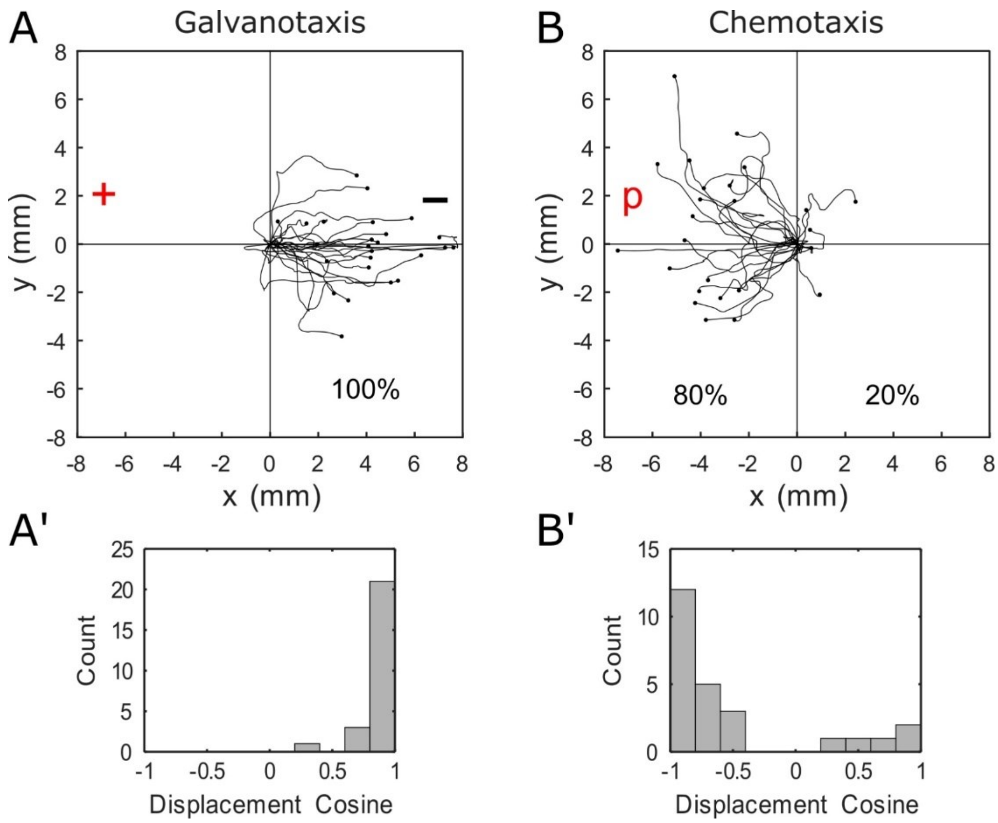

Figure S3. Control tests to check if the exposure to an electric field could modify the chemotactic response of *Amoeba proteus* cytoplasts to a peptide gradient. **(A)** Migration behavior of 25 *Amoeba proteus* cytoplasts under galvanotactic conditions (electric field). All the cytoplasts showed a normal galvanotactic response to the electric field, consisting in a directional migration toward the cathode. **(B)** The same 25 cytoplasts were then exposed to a chemotactic stimulus (nFMLP peptide gradient). The majority of them displayed a normal chemotaxis response by migrating against the peptide gradient. **(A', B')** Displacement cosines for the trajectories in (A) and (B). These analyses are consistent with the results obtained in the experiments described in the manuscript, both for cells and cytoplasts, in the galvanotaxis and chemotaxis scenarios, thus indicating that exposure to the electric field did not alter the cytoplast's normal chemotactic response to the peptide gradient. Experimental duration was 34'10''. "p" indicates the location of the chemotactic peptide, "+" anode, "–" cathode. BEID: N=25, Er=10, Nr=1-4.

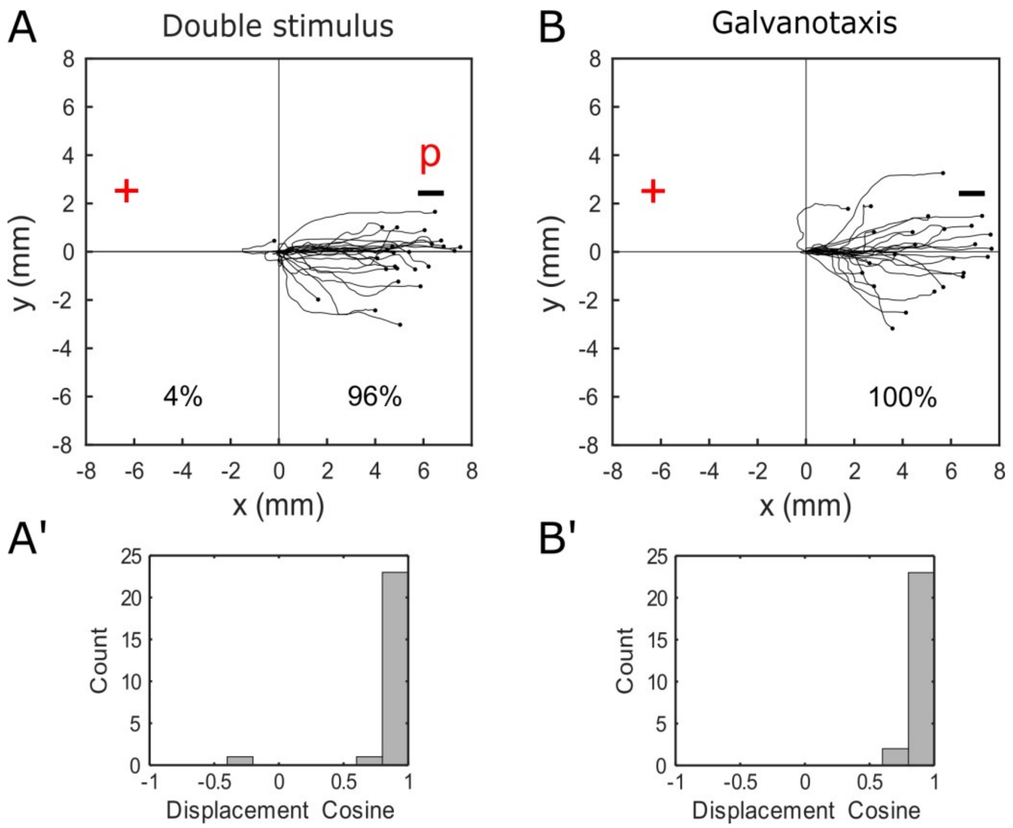

Figure S4. Control experiments to check if the simultaneous exposure to a peptide gradient and an electric field could modify the galvanotactic response of *Amoeba proteus* cytoplasts to an electric field. **(A)** Migration trajectories of 25 enucleated *Amoeba proteus* cytoplasts under simultaneous galvanotactic (electric field) and chemotactic (nFMLP peptide gradient) stimuli, where the peptide was placed on the cathode. All cytoplasts but one displayed clear directional migration towards the cathode. **(B)** The same 25 *Amoeba proteus* cytoplasts were then exposed to the galvanotactic stimulus, this time in the absence of peptide. Under this second scenario, all the cytoplasts showed clear directionality towards the cathode in their migration trajectories. **(A', B')** Displacement cosines for the trajectories in (A) and (B). These results show that simultaneous exposure to a chemotactic peptide gradient and an electric field did not alter the cytoplast's galvanotactic response. Experimental duration was 34'10''. "p" indicates the location of the chemotactic peptide, "+" anode, "–" cathode. BEID: N=25, Er=9, Nr=1-4.

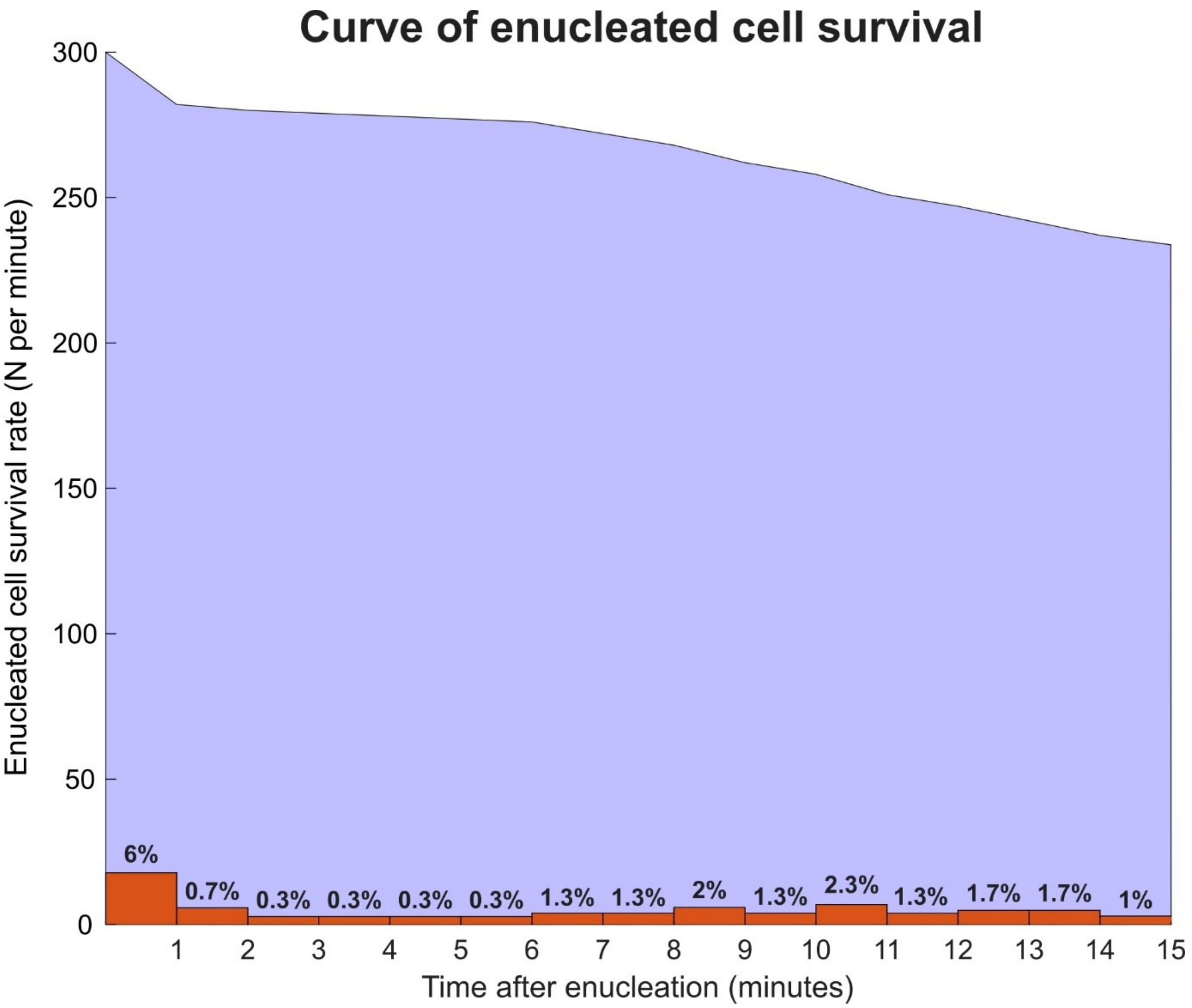

Figure S5. The curve represents the enucleated cells survival dynamic during the first 15 minutes after the enucleation. The histogram shows the number of cells that became acquiescent and stopped moving during the same period, and the relative percentage of the total 300 enucleated cells studied. It can be observed that 18 cells died within the first minute (6.00%), and all but 20 cells (6.67%) were able to acquire normal shape, adhesion, and movements by minute 2.

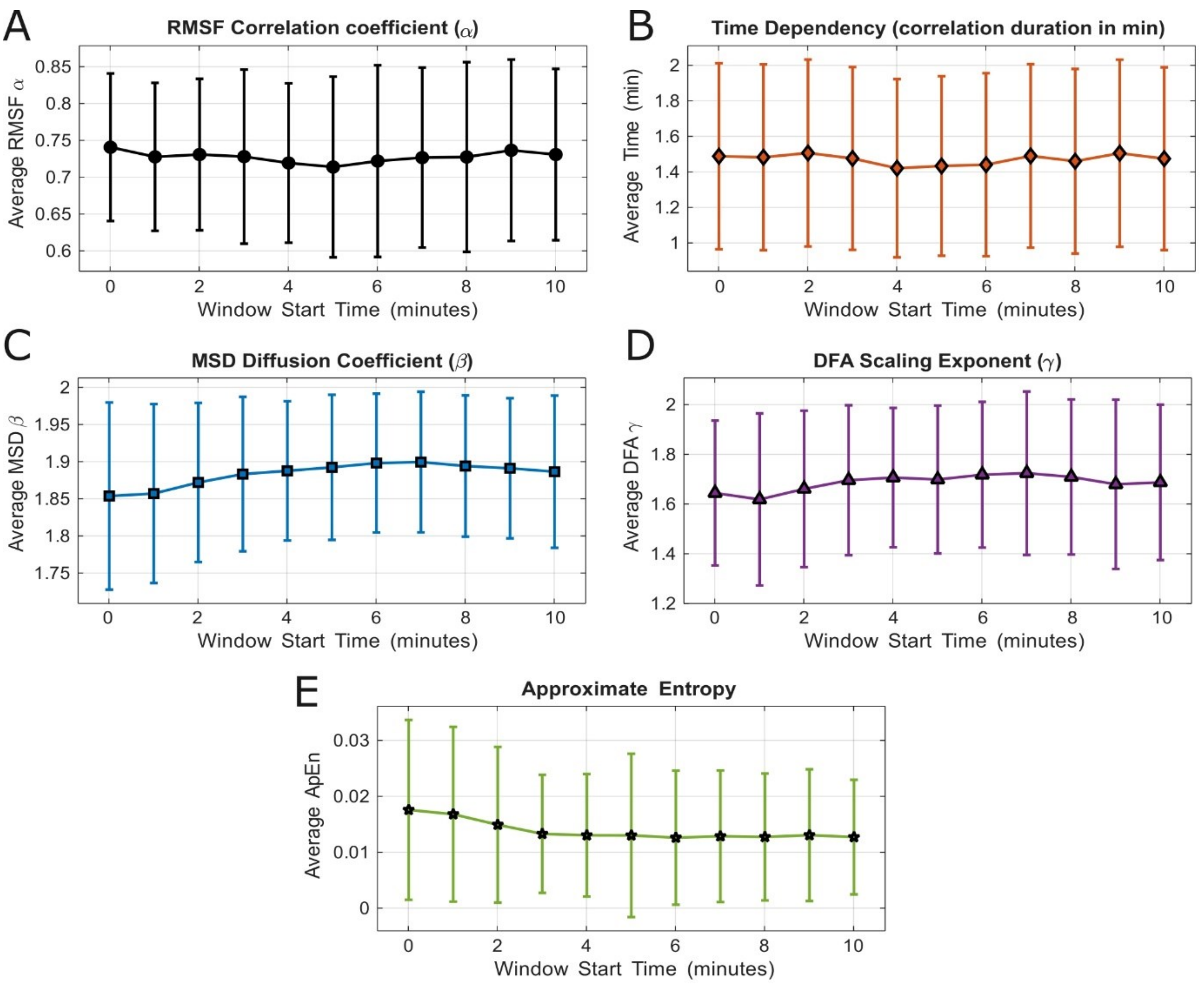

Figure S6. Analysis of 280 cellular migratory displacements of enucleated cells during the first 15 minutes after the recovery time. Trajectories were studied using a 5-minute sliding window (a moving time frame applied sequentially), advanced in 1-minute steps (Table 1). **(A–E)** Each panel shows the mean and standard deviation of a specific metric computed within each sliding window: **(A)** RMSF correlation coefficient (α); **(B)** time dependency (correlation times); **(C)** MSD diffusion coefficient (β); **(D)** DFA scaling exponent (γ); **(E)** Approximate Entropy. The X-axis represents the start time (in minutes) of each window (e.g., '0' corresponds to 0–5 min, '10' to 10–15 min). The standard deviation relative to the means was nearly zero in all cases (0.008, 0.029, 0.016, 0.033, and 0.002, respectively), indicating that after the recovery time there is a functional coherence in practically all the enucleated cell migratory trajectories analyzed. A total 280 enucleated cells were quantitatively studied, since 20 of the 300 cells were excluded due to death during the recovery time (see Figure 4).

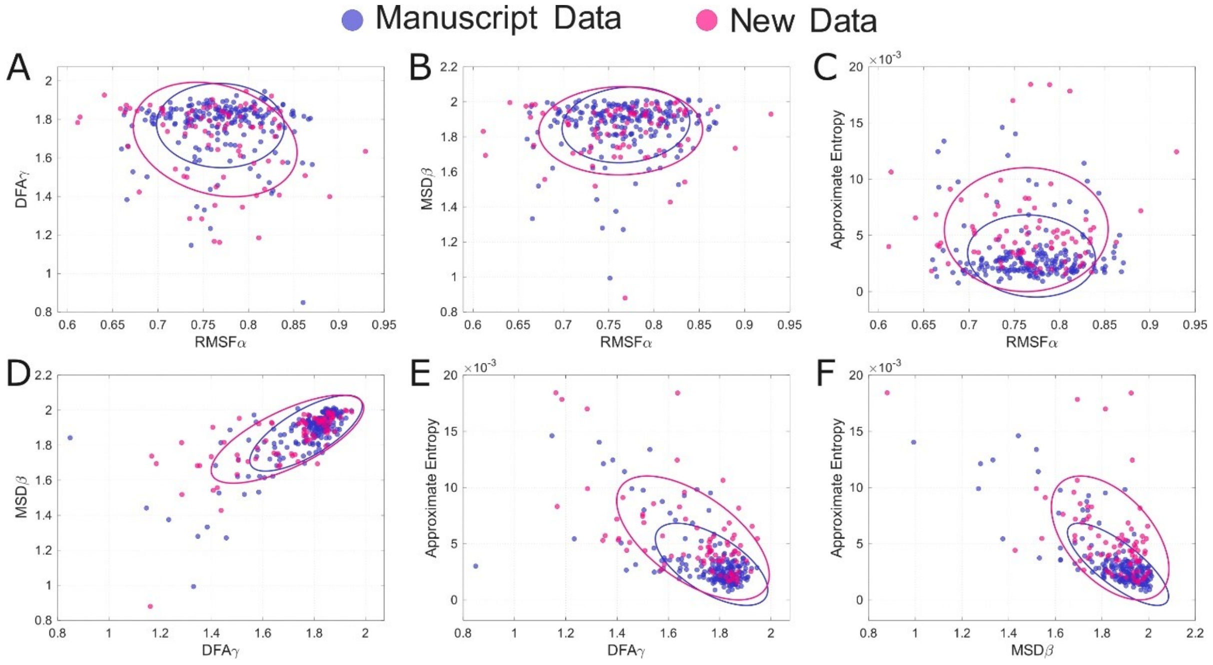

Figure S7. Analysis of the 280 migratory trajectories of enucleated cells over a 34’10’’ period, after the recovery time. **(A–F)** We have calculated four key non-linear metrics (RMSF correlation coefficient (α), MSD diffusion coefficient (β), Approximate Entropy, and DFA scaling exponent (γ)) (see Table 2). The results were consistent with those reported in the original manuscript and also corroborate that there is a functional coherence in the migratory trajectories of the enucleated cells analyzed. A total 280 enucleated cells were quantitatively studied, since 20 of the 300 cells were excluded due to death during the recovery time (see Figure 4).

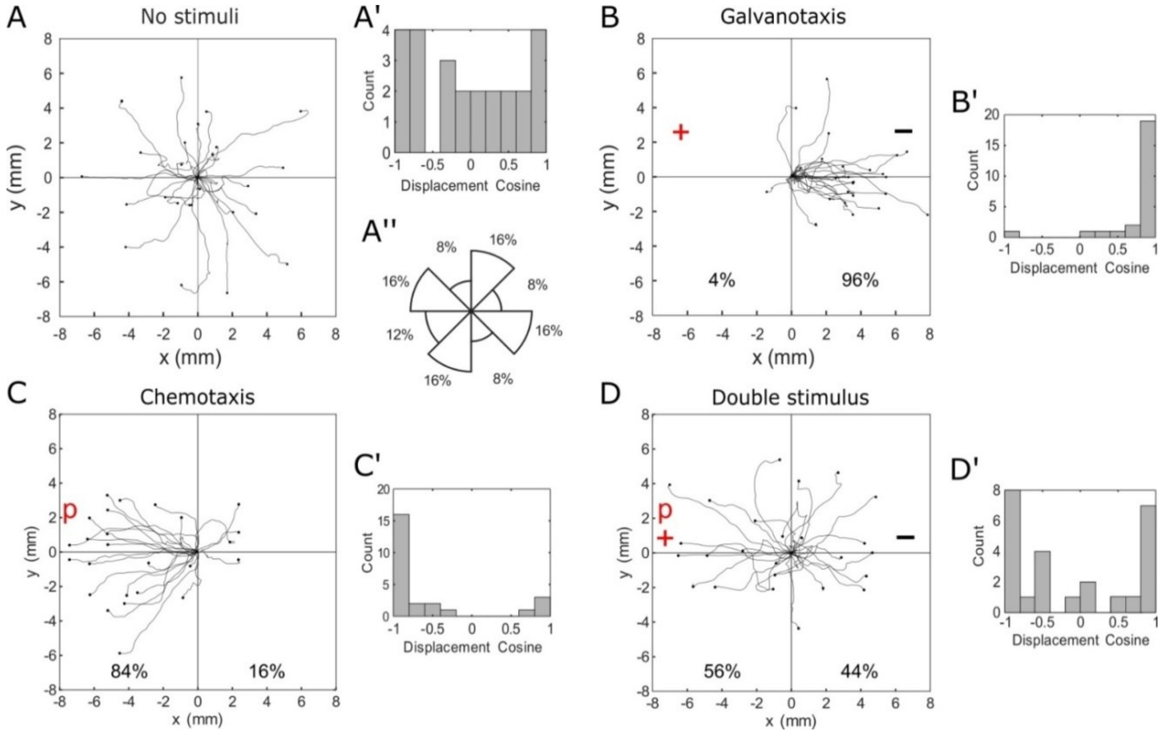

Figure S8. Migratory response of non-enucleated cells with a small portion of their cytoplasm removed, under the four experimental scenarios considered. **(A)** Migration trajectories in the absence of stimuli. **(A')** Displacement cosines corresponding to the trajectories in (A). **(A'')** The direction of cellular displacement in (A) is displayed as the percentage of cells that moved towards each of eight 45° sections within the experimental glass chamber. **(B)** Migration trajectories under an electric field. **(B')** Displacement cosines corresponding to the trajectories in (B). **(C)** Migration trajectories under a chemotactic peptide gradient. **(C')** Displacement cosines corresponding to the trajectories in (C). **(D)** Migration trajectories under simultaneous galvanotactic (electric field) and chemotactic (peptide gradient) stimuli. **(D')** Displacement cosines corresponding to the trajectories in (D). The results of Wilcoxon rank-sum tests comparing the displacement cosines of trajectories of enucleated cells and non-enucleated cells with a small portion of their cytoplasm removed show no significant differences under any of the four experimental scenarios considered (p-values: 0.422 for the absence of stimuli, 0.415 for galvanotaxis, 0.245 for chemotaxis and 0.525 for the double stimulus). Similarly, the results of Wilcoxon rank-sum tests comparing the displacement cosines of intact non-enucleated cells and non-enucleated cells with a small portion of their cytoplasm removed also show no significant differences under any of these four experimental scenarios considered (p-values: 0.354 for the absence of stimuli, 0.278 for galvanotaxis, 0.060 for chemotaxis and 0.62 for the double stimulus). These results indicate that there are no significant differences in the migratory responses between non-enucleated cells with a small portion of their cytoplasm removed, and both enucleated and intact non-enucleated cells, under the four experimental conditions considered. Experimental duration was 34'10''. "p" indicates the location of the chemotactic peptide, "+" anode, "–" cathode. BEID: N=25, Er=10, Nr=1-4 (No stimuli); N=25, Er=11, Nr=1-4 (Galvanotaxis); N=25, Er=11, Nr=1-4 (Chemotaxis); N=25, Er=9, Nr=1-4 (Double stimulus).

Table S1

#	Cells				Cytoplasts			
	Sc1	Sc2	Sc3	Sc4	Sc1	Sc2	Sc3	Sc4
1	0.742	0.725	0.652	0.769	0.672	0.667	0.757	0.838
2	0.781	0.761	0.75	0.738	0.766	0.819	0.78	0.776
3	0.824	0.737	0.742	0.798	0.666	0.726	0.774	0.781
4	0.777	0.749	0.76	0.709	0.827	0.772	0.714	0.772
5	0.822	0.79	0.743	0.805	0.764	0.688	0.732	0.781
6	0.868	0.739	0.751	0.737	0.813	0.757	0.682	0.811
7	0.678	0.777	0.795	0.679	0.785	0.79	0.72	0.768
8	0.703	0.652	0.628	0.754	0.751	0.758	0.745	0.751
9	0.606	0.775	0.725	0.696	0.69	0.693	0.765	0.763
10	0.853	0.633	0.736	0.757	0.772	0.741	0.795	0.696
11	0.651	0.765	0.685	0.744	0.781	0.818	0.797	0.815
12	0.73	0.742	0.702	0.797	0.738	0.784	0.743	0.795
13	0.805	0.711	0.739	0.733	0.737	0.85	0.87	0.794
14	0.661	0.735	0.636	0.701	0.86	0.826	0.786	0.66
15	0.676	0.671	0.777	0.783	0.733	0.804	0.754	0.698
16	0.69	0.718	0.727	0.8	0.818	0.744	0.734	0.848
17	0.753	0.69	0.763	0.659	0.744	0.711	0.75	0.796
18	0.725	0.661	0.759	0.768	0.817	0.849	0.813	0.714
19	0.738	0.744	0.597	0.666	0.754	0.729	0.806	0.797
20	0.664	0.659	0.693	0.83	0.86	0.795	0.851	0.776
21	0.8	0.691	0.748	0.69	0.714	0.767	0.775	0.817
22	0.801	0.624	0.782	0.713	0.803	0.835	0.823	0.75
23	0.719	0.697	0.688	0.678	0.732	0.802	0.759	0.741
24	0.81	0.716	0.767	0.7	0.802	0.788	0.758	0.807
25	0.728	0.641	0.698	0.832	0.778	0.741	0.838	0.705
26	0.784	0.766	0.79	0.712	0.737	0.785	0.831	0.756
27	0.725	0.744	0.785	0.762	0.688	0.8	0.738	0.772
28	0.751	0.747	0.767	0.823	0.742	0.78	0.822	0.733
29	0.756	0.667	0.702	0.722	0.814	0.769	0.816	0.694
30	0.753	0.684	0.65	0.715	0.785	0.775	0.849	0.815
31	0.704	0.751	0.719	0.753	0.827	0.774	0.761	0.731
32	0.651	0.713	0.616	0.702	0.774	0.664	0.731	0.737
33	0.771	0.841	0.71	0.713	0.766	0.81	0.814	0.792
34	0.799	0.626	0.742	0.762	0.663	0.714	0.766	0.776
35	0.807	0.827	0.696	0.793	0.778	0.754	0.705	0.723
36	0.78	0.617	0.763	0.745	0.755	0.733	0.695	0.76
37	0.737	0.709	0.695	0.769	0.826	0.778	0.746	0.719
38	0.769	0.738	0.756	0.756	0.832	0.787	0.861	0.735
39	0.792	0.765	0.73	0.704	0.806	0.768	0.742	0.766
40	0.709	0.73	0.766	0.739	0.764	0.755	0.733	0.786
41	0.679	0.662	0.779	0.751	0.714	0.819	0.84	0.694
42	0.721	0.68	0.71	0.776	0.801	0.788	0.742	0.745
43	0.775	0.748	0.729	0.727	0.83	0.78	0.794	0.771
44	0.828	0.759	0.749	0.742	0.74	0.767	0.717	0.686
45	0.873	0.721	0.741	0.716	0.797	0.791	0.866	0.807
46	0.725	0.747	0.723	0.744	0.806	0.767	0.701	0.756
47	0.853	0.738	0.7	0.733	0.827	0.709	0.765	0.671
48	0.851	0.669	0.726	0.755	0.719	0.81	0.792	0.811
49	0.684	0.673	0.741	0.743	0.782	0.78	0.747	0.758
50	0.68	0.732	0.775	0.796	0.843	0.746	0.736	0.868

Table S1. RMSF scaling exponent α values of the 400 experimental amoeba trajectories.

Table S2

#	Cells				Cytoplasts			
	Sc1	Sc2	Sc3	Sc4	Sc1	Sc2	Sc3	Sc4
1	0.44	0.51	0.435	0.449	0.536	0.431	0.41	0.484
2	0.428	0.48	0.537	0.517	0.394	0.515	0.365	0.431
3	0.445	0.493	0.443	0.512	0.411	0.524	0.414	0.513
4	0.415	0.518	0.455	0.574	0.497	0.404	0.466	0.553
5	0.529	0.403	0.497	0.446	0.481	0.517	0.543	0.461
6	0.462	0.43	0.503	0.469	0.473	0.433	0.468	0.505
7	0.405	0.402	0.44	0.429	0.396	0.46	0.387	0.445
8	0.443	0.46	0.379	0.474	0.456	0.535	0.414	0.525
9	0.482	0.561	0.401	0.512	0.527	0.47	0.622	0.514
10	0.548	0.535	0.52	0.487	0.448	0.475	0.507	0.521
11	0.553	0.483	0.496	0.615	0.533	0.405	0.439	0.454
12	0.468	0.408	0.467	0.52	0.521	0.469	0.506	0.536
13	0.464	0.451	0.447	0.498	0.437	0.474	0.55	0.524
14	0.502	0.444	0.583	0.447	0.532	0.509	0.433	0.43
15	0.422	0.446	0.563	0.405	0.569	0.416	0.487	0.54
16	0.477	0.496	0.505	0.53	0.561	0.467	0.549	0.471
17	0.427	0.454	0.465	0.459	0.481	0.421	0.438	0.504
18	0.481	0.503	0.493	0.49	0.405	0.506	0.502	0.402
19	0.512	0.471	0.497	0.523	0.44	0.387	0.474	0.459
20	0.521	0.406	0.438	0.436	0.488	0.48	0.425	0.418
21	0.4	0.426	0.405	0.558	0.373	0.454	0.533	0.47
22	0.385	0.405	0.504	0.462	0.436	0.484	0.534	0.401
23	0.436	0.471	0.47	0.466	0.515	0.502	0.509	0.573
24	0.431	0.485	0.505	0.434	0.495	0.497	0.524	0.504
25	0.492	0.477	0.461	0.442	0.503	0.442	0.426	0.45
26	0.587	0.377	0.429	0.456	0.456	0.409	0.449	0.503
27	0.39	0.441	0.447	0.612	0.529	0.469	0.469	0.468
28	0.447	0.498	0.488	0.5	0.437	0.432	0.558	0.451
29	0.473	0.504	0.446	0.42	0.5	0.488	0.428	0.482
30	0.522	0.449	0.517	0.594	0.609	0.486	0.425	0.44
31	0.471	0.423	0.498	0.41	0.457	0.514	0.499	0.431
32	0.448	0.489	0.503	0.532	0.477	0.398	0.466	0.495
33	0.517	0.416	0.408	0.429	0.519	0.404	0.598	0.442
34	0.491	0.395	0.508	0.48	0.476	0.48	0.431	0.538
35	0.529	0.421	0.519	0.545	0.395	0.53	0.457	0.495
36	0.429	0.454	0.54	0.484	0.397	0.531	0.58	0.522
37	0.494	0.402	0.449	0.473	0.411	0.487	0.444	0.469
38	0.484	0.458	0.468	0.457	0.48	0.567	0.548	0.486
39	0.47	0.463	0.456	0.484	0.396	0.421	0.453	0.508
40	0.468	0.447	0.443	0.41	0.486	0.505	0.412	0.453
41	0.47	0.4	0.466	0.495	0.453	0.521	0.449	0.45
42	0.512	0.51	0.468	0.427	0.422	0.431	0.456	0.406
43	0.475	0.599	0.403	0.475	0.472	0.398	0.503	0.508
44	0.512	0.415	0.48	0.385	0.468	0.491	0.455	0.407
45	0.486	0.371	0.485	0.541	0.439	0.515	0.543	0.425
46	0.444	0.477	0.444	0.449	0.47	0.521	0.443	0.546
47	0.48	0.421	0.454	0.495	0.433	0.423	0.428	0.51
48	0.539	0.45	0.444	0.487	0.47	0.451	0.464	0.401
49	0.538	0.509	0.436	0.408	0.451	0.491	0.457	0.445
50	0.467	0.425	0.455	0.438	0.503	0.466	0.431	0.407

Table S2. RMSF scaling exponent α values of the 400 shuffled amoeba trajectories.

Table S3

#	Cells				Cytoplasts			
	Sc1	Sc2	Sc3	Sc4	Sc1	Sc2	Sc3	Sc4
1	15.625	4.167	6.25	8.333	9.375	8.333	4.167	3.125
2	5.208	7.292	9.375	7.292	9.375	16.667	4.167	4.167
3	13.542	3.125	3.125	3.125	14.583	16.667	9.375	3.125
4	14.583	15.625	7.292	9.375	2.083	11.458	9.375	9.375
5	11.458	6.25	15.625	7.292	8.333	6.25	12.5	3.125
6	16.667	13.542	12.5	17.708	16.667	11.458	6.25	5.208
7	5.208	16.667	5.208	10.417	7.292	10.417	15.625	8.333
8	6.25	5.208	15.625	15.625	6.25	13.542	13.542	8.333
9	16.667	17.708	6.25	17.708	4.167	9.375	16.667	4.167
10	16.667	13.542	11.458	13.542	5.208	12.5	8.333	5.208
11	5.208	3.125	13.542	11.458	4.167	8.333	12.5	9.375
12	5.208	3.125	12.5	8.333	8.333	6.25	15.625	9.375
13	4.167	2.083	17.708	12.5	4.167	13.542	8.333	4.167
14	12.5	9.375	5.208	13.542	8.333	9.375	13.542	12.5
15	7.292	2.083	14.583	9.375	5.208	13.542	7.292	10.417
16	4.167	10.417	7.292	6.25	8.333	5.208	3.125	7.292
17	3.125	5.208	4.167	10.417	13.542	15.625	7.292	6.25
18	4.167	15.625	11.458	12.5	13.542	16.667	16.667	14.583
19	15.625	2.083	17.708	2.083	5.208	8.333	9.375	4.167
20	12.5	4.167	10.417	13.542	5.208	7.292	11.458	8.333
21	13.542	5.208	9.375	13.542	8.333	14.583	5.208	6.25
22	13.542	6.25	8.333	8.333	2.083	6.25	13.542	15.625
23	12.5	15.625	7.292	10.417	8.333	7.292	6.25	10.417
24	15.625	13.542	7.292	14.583	15.625	7.292	7.292	15.625
25	8.333	15.625	4.167	15.625	7.292	10.417	6.25	12.5
26	16.667	15.625	6.25	7.292	13.542	5.208	6.25	6.25
27	13.542	6.25	10.417	17.708	17.708	6.25	9.375	6.25
28	8.333	7.292	6.25	10.417	3.125	9.375	9.375	5.208
29	4.167	12.5	3.125	6.25	10.417	9.375	12.5	8.333
30	7.292	7.292	18.75	13.542	13.542	15.625	12.5	12.5
31	10.417	7.292	4.167	11.458	12.5	5.208	11.458	12.5
32	17.708	11.458	10.417	16.667	14.583	10.417	5.208	7.292
33	15.625	17.708	13.542	16.667	5.208	16.667	7.292	3.125
34	8.333	16.667	9.375	7.292	10.417	7.292	10.417	7.292
35	17.708	16.667	10.417	18.75	9.375	11.458	8.333	14.583
36	7.292	17.708	15.625	8.333	9.375	6.25	6.25	5.208
37	6.25	15.625	11.458	13.542	3.125	8.333	10.417	6.25
38	15.625	11.458	3.125	10.417	7.292	8.333	4.167	8.333
39	15.625	8.333	10.417	8.333	3.125	8.333	10.417	6.25
40	13.542	14.583	15.625	11.458	8.333	8.333	10.417	10.417
41	7.292	6.25	9.375	16.667	4.167	6.25	4.167	5.208
42	14.583	12.5	9.375	4.167	14.583	9.375	7.292	8.333
43	3.125	14.583	4.167	3.125	7.292	8.333	3.125	5.208
44	7.292	17.708	12.5	8.333	11.458	6.25	6.25	12.5
45	3.125	11.458	11.458	5.208	10.417	6.25	4.167	15.625
46	6.25	16.667	6.25	16.667	17.708	16.667	9.375	14.583
47	17.708	16.667	8.333	14.583	15.625	17.708	5.208	13.542
48	17.708	14.583	8.333	6.25	6.25	5.208	4.167	8.333
49	9.375	13.542	11.458	4.167	10.417	11.458	5.208	10.417
50	13.542	12.5	10.417	14.583	4.167	9.375	8.333	7.292

Table S3. Long-range correlation span (memory time) in minutes for the 400 experimental amoeba trajectories.

Table S4

#	Cells				Cytoplasts			
	Sc1	Sc2	Sc3	Sc4	Sc1	Sc2	Sc3	Sc4
1	1.96	1.955	1.994	1.775	1.519	1.742	1.928	2.013
2	1.981	1.921	1.998	1.956	1.271	1.993	1.858	1.995
3	1.972	1.934	1.719	1.902	1.333	1.975	1.947	1.908
4	1.936	1.95	1.974	1.622	1.527	1.97	1.86	1.968
5	1.971	1.776	1.931	1.796	1.854	1.621	1.844	2.004
6	1.929	1.982	1.834	1.801	1.89	1.911	1.996	1.935
7	2.001	1.923	1.893	1.851	1.715	1.932	1.938	1.989
8	1.971	1.887	1.979	1.934	0.993	1.815	1.892	1.976
9	1.97	1.842	1.934	1.826	1.894	1.882	1.908	1.923
10	1.948	1.987	1.867	1.902	1.955	1.774	1.9	1.944
11	1.993	1.984	1.952	1.951	1.991	1.998	1.804	1.925
12	1.945	1.958	1.956	1.855	1.847	1.891	1.279	1.92
13	1.957	1.925	1.973	1.91	1.442	1.89	1.973	1.614
14	2.01	1.933	1.957	1.984	1.842	1.921	1.803	1.787
15	1.899	1.984	1.82	1.979	1.97	1.991	1.91	1.759
16	1.938	1.878	1.615	1.965	1.944	1.947	2.001	1.876
17	1.706	1.938	1.757	1.985	1.914	1.955	1.988	1.841
18	1.604	1.811	1.909	1.968	1.97	1.922	1.944	1.869
19	1.813	1.877	1.936	1.996	1.974	1.882	1.862	1.914
20	1.613	1.992	1.957	1.995	1.933	1.888	2.009	1.818
21	1.931	1.899	1.826	1.963	1.997	1.911	1.818	1.807
22	1.879	1.968	1.962	1.992	2.001	1.922	1.887	1.809
23	1.81	1.949	1.95	1.909	1.622	1.83	1.81	1.818
24	1.928	1.971	1.791	1.853	1.697	1.935	1.982	1.623
25	1.926	1.983	1.934	1.834	1.757	1.922	2.004	1.972
26	2.006	1.973	1.888	1.893	1.859	1.841	1.898	1.812
27	1.788	1.955	1.806	1.965	1.988	1.808	1.881	1.903
28	1.949	1.928	1.94	1.828	1.684	1.94	1.911	1.813
29	1.799	1.955	1.922	1.809	1.86	1.803	1.917	1.938
30	1.414	1.915	1.9	1.851	1.919	1.891	1.921	1.875
31	1.978	1.904	1.935	1.786	1.754	1.814	1.982	1.738
32	1.955	1.955	1.865	1.833	1.957	1.974	1.903	1.93
33	2.003	1.937	1.903	1.954	1.82	1.891	1.989	1.926
34	1.93	1.944	1.876	1.679	1.938	1.89	1.911	1.96
35	1.971	1.916	1.927	1.901	1.935	1.89	1.909	1.726
36	1.92	1.992	1.926	1.612	1.748	1.94	1.955	1.858
37	1.958	1.971	1.841	1.841	1.684	1.78	1.622	1.939
38	1.947	1.989	1.973	1.862	1.844	1.955	1.858	1.98
39	1.832	2.011	1.788	1.888	1.898	1.853	1.909	1.788
40	1.978	1.981	1.943	1.836	1.976	1.988	1.967	1.921
41	1.893	1.99	1.952	1.743	1.965	1.918	1.942	1.931
42	1.811	1.994	1.941	1.777	1.96	1.926	1.937	2.007
43	1.965	1.931	1.963	1.769	1.721	1.974	1.949	1.908
44	1.769	1.689	1.983	1.81	1.932	1.986	2.007	1.861
45	1.799	1.969	2.01	1.891	1.934	1.948	1.884	1.877
46	1.891	1.951	1.938	1.828	1.948	1.966	1.916	1.9
47	1.938	1.973	1.911	1.786	1.948	1.897	1.892	1.826
48	1.924	1.977	1.987	1.874	1.906	1.895	2.008	1.718
49	1.872	1.982	1.978	1.416	1.897	1.532	1.949	1.375
50	2.006	1.989	1.913	1.894	1.735	1.879	1.922	1.879

Table S4. MSD diffusion exponent β values of the 400 experimental amoeba trajectories.

Table S5

#	Cells				Cytoplasts			
	Sc1	Sc2	Sc3	Sc4	Sc1	Sc2	Sc3	Sc4
1	0	0.002	-0.001	-0.001	0	0.001	0	0
2	-0.001	-0.001	0.001	-0.001	-0.001	-0.002	-0.002	-0.001
3	0.001	0.001	-0.002	0	-0.002	0.001	-0.004	0.003
4	0.002	-0.001	0	-0.003	0.001	0	-0.002	0.003
5	-0.001	0.001	-0.002	0.001	0	-0.002	0.001	-0.001
6	-0.004	0	0	-0.003	0	-0.001	-0.001	0.001
7	0	0.001	0.002	0.001	-0.004	0	0.002	-0.001
8	-0.001	-0.003	-0.001	0.001	0	-0.002	0.002	0.003
9	0	0	-0.003	-0.001	-0.001	0.001	-0.002	0.002
10	-0.003	0.001	-0.001	0	0.002	-0.002	0.005	-0.002
11	0.002	0	0	-0.001	-0.004	-0.004	0.002	-0.001
12	0	0.001	0.004	0	0	0.004	-0.001	-0.003
13	0.003	-0.001	-0.002	0	0.001	-0.002	0	0.003
14	0	0	-0.001	0.001	0.002	-0.003	-0.002	0.001
15	-0.002	-0.002	0	-0.002	-0.001	0	0	0.002
16	0.001	0	-0.002	0	-0.003	0	-0.003	0
17	0.001	-0.001	0	0.001	0.003	0	-0.001	-0.001
18	-0.001	0.002	-0.001	0.001	-0.001	0.001	-0.002	0.001
19	-0.003	-0.001	0	-0.003	0.002	0.001	-0.001	-0.001
20	0	0	-0.001	-0.001	0.002	0	0.001	0
21	-0.002	0.002	-0.004	0.001	-0.004	0	0.003	0.001
22	0.001	-0.003	-0.001	0.001	-0.001	0.011	0.002	0
23	0.001	0	0	-0.001	0	0	-0.004	0
24	0	-0.001	0.001	-0.001	0	0.001	-0.002	0.001
25	-0.002	-0.001	0.001	0.002	0.002	0.001	-0.001	0.002
26	-0.001	0	0.003	-0.001	0	-0.002	-0.004	-0.003
27	0.001	-0.002	0.003	0	-0.001	-0.002	0.002	0.001
28	-0.004	0	0.002	0.002	0.003	-0.002	0.001	0.002
29	-0.001	-0.001	0	0	0	-0.001	0	-0.002
30	0.002	0	-0.001	-0.002	0	-0.003	0.001	0.001
31	-0.001	0	-0.002	0.001	0	-0.001	-0.002	0
32	0	0.001	0.004	0	-0.001	0	0	0
33	0.001	0.002	0.001	0.001	-0.001	-0.001	0.002	0.003
34	0	-0.001	0.003	-0.002	0.001	0	-0.003	-0.002
35	0.002	-0.001	0.003	0	0	-0.001	0.003	0.002
36	-0.001	0	0.001	-0.002	0.004	-0.001	-0.002	0
37	0.003	0.002	0	0.001	-0.002	0.002	-0.002	0
38	0.001	0.001	0	0.001	-0.002	-0.004	-0.003	-0.001
39	0.001	0.002	0.005	-0.002	-0.002	0.002	-0.001	0
40	0	0.001	-0.001	0.002	0	0.002	0.002	-0.004
41	-0.001	0.001	0	0.001	0.001	0	-0.001	-0.001
42	0.001	0	0.002	0.001	0.001	-0.002	0	-0.002
43	0	0	-0.002	-0.001	0.001	0.002	0.001	0.001
44	-0.001	-0.004	-0.001	0	-0.001	0	-0.001	-0.001
45	0	0	0	-0.001	-0.002	0.003	-0.002	-0.002
46	0	-0.001	-0.003	-0.002	0.001	0	0	-0.001
47	0.003	0.001	0.002	0.001	-0.002	-0.001	0	0
48	0.002	0.003	0.002	0	-0.001	0.001	-0.001	0.005
49	-0.001	0	0	0	0.001	-0.001	-0.001	-0.003
50	0.001	-0.001	-0.001	0.003	0.005	0.001	-0.001	0

Table S5. MSD diffusion exponent β values of the 400 shuffled amoeba trajectories.

Table S6

#	Cells				Cytoplasts			
	Sc1	Sc2	Sc3	Sc4	Sc1	Sc2	Sc3	Sc4
1	0.002	0.002	0.001	0.002	0.013	0.009	0.002	0.002
2	0.001	0.002	0.001	0.002	0.01	0.002	0.003	0.001
3	0.001	0.002	0.007	0.002	0.012	0.002	0.003	0.003
4	0.002	0.002	0.003	0.007	0.011	0.002	0.003	0.004
5	0.001	0.002	0.002	0.004	0.002	0.01	0.002	0.001
6	0.002	0.001	0.003	0.002	0.002	0.003	0.002	0.001
7	0.002	0.002	0.003	0.006	0.005	0.002	0.002	0.002
8	0.002	0.002	0.002	0.001	0.014	0.003	0.002	0.002
9	0.002	0.003	0.002	0.002	0.003	0.002	0.002	0.002
10	0.002	0.001	0.003	0.002	0.002	0.003	0.002	0.002
11	0.001	0.001	0.002	0.002	0.003	0.003	0.003	0.002
12	0.002	0.002	0.001	0.002	0.003	0.003	0.012	0.002
13	0.003	0.002	0.001	0.002	0.015	0.002	0.003	0.008
14	0.001	0.002	0.002	0.001	0.003	0.002	0.004	0.003
15	0.003	0.001	0.002	0.001	0.003	0.001	0.003	0.002
16	0.003	0.002	0.003	0.003	0.003	0.002	0.003	0.002
17	0.004	0.002	0.002	0.001	0.003	0.002	0.002	0.003
18	0.009	0.002	0.002	0.001	0.002	0.002	0.002	0.002
19	0.004	0.002	0.002	0.001	0.003	0.003	0.004	0.002
20	0.004	0.001	0.002	0.002	0.003	0.003	0.002	0.003
21	0.002	0.002	0.004	0.001	0.002	0.002	0.003	0.002
22	0.002	0.001	0.001	0.002	0.002	0.003	0.003	0.002
23	0.002	0.001	0.002	0.003	0.005	0.002	0.003	0.004
24	0.002	0.001	0.003	0.003	0.003	0.002	0.003	0.004
25	0.002	0.001	0.002	0.002	0.003	0.002	0.002	0.001
26	0.002	0.001	0.003	0.002	0.002	0.003	0.01	0.003
27	0.005	0.001	0.003	0.002	0.001	0.002	0.003	0.001
28	0.002	0.001	0.002	0.002	0.003	0.002	0.002	0.003
29	0.002	0.003	0.003	0.004	0.002	0.002	0.002	0.006
30	0.014	0.003	0.003	0.002	0.002	0.002	0.003	0.002
31	0.002	0.002	0.003	0.002	0.005	0.01	0.002	0.008
32	0.001	0.002	0.003	0.003	0.001	0.001	0.003	0.003
33	0.001	0.001	0.002	0.001	0.002	0.003	0.002	0.003
34	0.001	0.002	0.002	0.004	0.002	0.003	0.005	0.003
35	0.003	0.002	0.003	0.002	0.002	0.002	0.003	0.004
36	0.005	0.001	0.002	0.007	0.004	0.002	0.002	0.002
37	0.002	0.001	0.002	0.003	0.005	0.004	0.004	0.001
38	0.002	0.001	0.002	0.003	0.004	0.003	0.004	0.001
39	0.002	0.002	0.003	0.002	0.003	0.002	0.003	0.007
40	0.002	0.002	0.002	0.003	0.002	0.002	0.001	0.002
41	0.002	0.001	0.002	0.003	0.003	0.002	0.002	0.002
42	0.003	0.001	0.002	0.005	0.003	0.002	0.002	0.001
43	0.002	0.002	0.001	0.005	0.007	0.002	0.002	0.002
44	0.006	0.008	0.002	0.004	0.002	0.001	0.002	0.004
45	0.006	0.002	0.001	0.003	0.002	0.002	0.005	0.002
46	0.003	0.002	0.002	0.003	0.003	0.001	0.002	0.004
47	0.002	0.001	0.003	0.003	0.002	0.002	0.004	0.004
48	0.001	0.001	0.002	0.002	0.002	0.002	0.003	0.008
49	0.003	0.001	0.003	0.007	0.002	0.004	0.002	0.005
50	0.001	0.001	0.002	0.002	0.009	0.003	0.002	0.002

Table S6. Approximate Entropy (ApEn) values for the 400 experimental amoeba trajectories.

Table S7

#	Cells				Cytoplasts			
	Sc1	Sc2	Sc3	Sc4	Sc1	Sc2	Sc3	Sc4
1	2.09	2.067	2.088	1.9	1.942	2.109	2.062	1.893
2	2.081	2.091	2.047	1.924	2.013	1.858	1.735	2.058
3	2.005	2.081	1.945	2.018	2.122	1.909	1.965	1.94
4	2.029	2.074	1.955	1.943	1.759	1.964	1.946	1.941
5	1.941	1.926	1.716	1.957	1.878	1.96	1.664	2.07
6	1.976	2.05	1.881	1.916	1.478	1.957	2.069	2.053
7	2.081	2.042	1.961	2.053	1.702	1.849	1.701	1.993
8	2.105	1.932	2.044	1.923	1.98	1.793	1.681	1.79
9	2.072	1.725	2.03	1.792	1.905	2.003	1.577	2.017
10	1.878	2.092	1.922	2.051	2.07	1.945	1.826	2.02
11	2.079	2.087	2.109	1.919	1.914	1.752	1.736	1.995
12	2.084	2.083	2.104	2.02	1.909	1.725	2.037	1.786
13	1.92	2.108	2.075	2.004	1.921	1.532	1.683	1.743
14	2.062	2.021	1.916	2.028	1.298	1.83	1.704	1.975
15	2.103	2.094	1.553	1.967	1.825	1.937	1.824	1.813
16	2.027	2.033	1.997	1.75	1.811	2.072	1.966	1.793
17	1.92	2.128	1.945	2.08	1.943	1.893	2.035	1.825
18	1.99	1.853	1.872	1.967	1.824	1.691	1.846	1.856
19	1.612	2.09	2.063	2.104	1.985	1.69	1.635	2.017
20	2.022	2.066	2.081	1.854	1.618	1.818	1.802	1.724
21	1.943	2.055	1.912	2.086	1.959	1.954	1.948	1.951
22	1.679	2.092	2.067	2.044	1.969	1.837	1.744	1.601
23	1.966	2.065	2.089	1.967	1.725	1.772	1.851	1.896
24	1.742	2.042	1.997	1.825	1.69	2.019	1.825	1.471
25	1.925	2.023	2.067	1.511	1.731	2.04	1.682	1.996
26	1.946	2.044	1.998	1.921	1.844	1.804	1.881	1.763
27	2.029	1.945	1.937	1.986	2.1	1.948	1.682	2.018
28	2.099	2.055	1.996	1.804	2.059	2.015	1.807	1.929
29	2.017	2.047	2.055	1.797	1.809	1.843	1.914	2.029
30	2.016	2.066	2.104	1.95	2.021	1.872	1.812	1.648
31	1.95	2.083	2.034	1.778	1.619	1.78	1.932	1.944
32	2.077	1.998	1.934	2.014	1.993	2.074	2.01	1.933
33	1.99	1.754	1.98	2.1	1.979	1.914	1.959	2.022
34	1.9	2.091	1.907	1.929	2.078	1.906	1.9	1.688
35	1.927	1.853	1.926	2.017	1.934	1.631	1.991	1.962
36	1.993	2.111	1.895	1.964	1.927	2.061	2.1	2.035
37	2.013	2.081	1.964	1.483	1.957	1.583	1.803	2.074
38	2.039	2.013	2.066	1.967	1.451	1.825	1.575	1.996
39	1.907	1.992	1.808	1.892	1.927	1.769	1.915	1.843
40	1.961	2.04	1.944	1.908	1.966	1.95	2.089	1.71
41	2.056	2.084	2.023	1.814	1.99	1.969	1.943	2.092
42	1.913	2.069	2.077	1.761	1.819	1.764	2.065	2.002
43	2.046	2.058	2.098	1.97	1.963	2.005	2.045	2.037
44	1.699	1.901	1.899	1.871	2.072	2.047	2.013	1.832
45	1.855	1.999	1.982	2.039	1.931	1.992	1.712	1.774
46	2.059	2.08	2.104	1.991	1.618	1.964	2.06	1.801
47	1.627	2.078	1.887	1.606	1.923	2.073	1.915	1.791
48	1.794	2.114	2.029	1.942	2.062	2.027	1.842	1.947
49	2.011	2.095	1.877	1.99	2.032	2.076	1.979	1.449
50	2.07	2.07	1.895	1.835	1.95	1.89	1.888	1.582

Table S7. Approximate Entropy (ApEn) values for the 400 shuffled amoeba trajectories

Table S8

#	Cells				Cytoplasts			
	Sc1	Sc2	Sc3	Sc4	Sc1	Sc2	Sc3	Sc4
1	1.842	1.824	1.838	1.714	1.528	1.662	1.792	1.876
2	1.826	1.8	1.839	1.87	1.458	1.905	1.669	1.84
3	1.831	1.825	1.637	1.858	1.383	1.852	1.741	1.784
4	1.801	1.793	1.835	1.645	1.429	1.844	1.806	1.892
5	1.83	1.713	1.844	1.722	1.803	1.502	1.827	1.849
6	1.692	1.847	1.606	1.628	1.85	1.874	1.854	1.817
7	1.85	1.841	1.795	1.749	1.665	1.836	1.855	1.847
8	1.821	1.797	1.852	1.807	1.33	1.436	1.642	1.879
9	1.824	1.788	1.844	1.618	1.844	1.791	1.853	1.807
10	1.839	1.834	1.839	1.774	1.817	1.58	1.811	1.776
11	1.835	1.827	1.804	1.833	1.832	1.945	1.795	1.781
12	1.828	1.818	1.814	1.74	1.768	1.89	1.347	1.72
13	1.886	1.795	1.827	1.79	1.146	1.817	1.566	1.606
14	1.85	1.727	1.848	1.847	0.849	1.737	1.852	1.646
15	1.786	1.816	1.808	1.861	1.781	1.863	1.854	1.806
16	1.877	1.723	1.614	1.625	1.908	1.81	1.87	1.826
17	1.761	1.785	1.789	1.854	1.812	1.83	1.834	1.847
18	1.569	1.706	1.872	1.841	1.898	1.832	1.779	1.833
19	1.731	1.773	1.828	1.848	1.879	1.711	1.89	1.853
20	1.646	1.855	1.822	1.886	1.574	1.852	1.736	1.728
21	1.819	1.807	1.629	1.819	1.887	1.831	1.671	1.738
22	1.817	1.811	1.823	1.853	1.862	1.772	1.472	1.815
23	1.779	1.778	1.819	1.85	1.544	1.545	1.644	1.605
24	1.822	1.816	1.732	1.689	1.763	1.817	1.901	1.638
25	1.846	1.826	1.85	1.368	1.832	1.823	1.878	1.861
26	1.875	1.771	1.832	1.844	1.794	1.799	1.711	1.729
27	1.747	1.844	1.71	1.822	1.834	1.784	1.844	1.836
28	1.793	1.782	1.854	1.699	1.653	1.836	1.836	1.794
29	1.744	1.827	1.86	1.651	1.684	1.817	1.806	1.778
30	1.313	1.788	1.78	1.735	1.787	1.869	1.844	1.817
31	1.862	1.786	1.843	1.809	1.781	1.764	1.888	1.758
32	1.836	1.839	1.815	1.739	1.858	1.85	1.786	1.875
33	1.88	1.835	1.813	1.79	1.76	1.831	1.91	1.794
34	1.852	1.817	1.819	1.627	1.824	1.816	1.728	1.897
35	1.865	1.818	1.908	1.725	1.811	1.899	1.842	1.679
36	1.771	1.828	1.85	1.444	1.651	1.807	1.824	1.766
37	1.806	1.824	1.642	1.508	1.416	1.819	1.588	1.774
38	1.819	1.849	1.836	1.846	1.859	1.924	1.816	1.87
39	1.788	1.889	1.592	1.854	1.835	1.814	1.845	1.694
40	1.888	1.849	1.824	1.618	1.853	1.883	1.781	1.858
41	1.764	1.858	1.788	1.732	1.856	1.823	1.757	1.811
42	1.747	1.848	1.8	1.593	1.85	1.836	1.817	1.885
43	1.844	1.813	1.816	1.76	1.627	1.865	1.811	1.8
44	1.695	1.615	1.806	1.839	1.797	1.862	1.852	1.797
45	1.688	1.869	1.855	1.79	1.82	1.815	1.801	1.879
46	1.824	1.799	1.814	1.733	1.831	1.848	1.818	1.828
47	1.84	1.832	1.732	1.821	1.85	1.807	1.76	1.767
48	1.821	1.817	1.854	1.828	1.779	1.814	1.844	1.505
49	1.816	1.825	1.9	1.592	1.78	1.584	1.801	1.233
50	1.849	1.834	1.857	1.792	1.576	1.842	1.906	1.805

Table S8. Detrended Fluctuation Analysis (DFA) scaling exponent γ values for the 400 experimental amoeba trajectories.

Table S9

#	Cells				Cytoplasts			
	Sc1	Sc2	Sc3	Sc4	Sc1	Sc2	Sc3	Sc4
1	0.425	0.482	0.507	0.481	0.347	0.539	0.529	0.302
2	0.352	0.509	0.516	0.513	0.505	0.35	0.421	0.576
3	0.415	0.401	0.312	0.555	0.42	0.569	0.303	0.55
4	0.6	0.527	0.414	0.429	0.303	0.546	0.461	0.524
5	0.551	0.304	0.503	0.421	0.375	0.619	0.557	0.456
6	0.526	0.355	0.384	0.518	0.484	0.303	0.51	0.576
7	0.445	0.566	0.421	0.46	0.475	0.544	0.377	0.509
8	0.524	0.549	0.442	0.455	0.529	0.604	0.554	0.282
9	0.355	0.663	0.608	0.479	0.443	0.41	0.551	0.281
10	0.508	0.511	0.476	0.461	0.592	0.45	0.551	0.507
11	0.332	0.305	0.408	0.482	0.505	0.608	0.427	0.513
12	0.539	0.495	0.422	0.466	0.558	0.472	0.603	0.539
13	0.466	0.496	0.33	0.354	0.552	0.562	0.589	0.584
14	0.53	0.445	0.422	0.596	0.657	0.505	0.496	0.419
15	0.467	0.434	0.497	0.361	0.611	0.454	0.547	0.366
16	0.456	0.467	0.389	0.344	0.421	0.472	0.664	0.406
17	0.276	0.569	0.397	0.39	0.469	0.418	0.496	0.53
18	0.62	0.634	0.554	0.478	0.602	0.516	0.325	0.473
19	0.537	0.471	0.38	0.507	0.419	0.48	0.484	0.487
20	0.462	0.416	0.531	0.364	0.533	0.646	0.35	0.537
21	0.629	0.429	0.387	0.467	0.694	0.573	0.455	0.46
22	0.315	0.597	0.428	0.353	0.343	0.444	0.548	0.36
23	0.317	0.476	0.445	0.344	0.388	0.52	0.534	0.689
24	0.553	0.563	0.445	0.41	0.346	0.529	0.572	0.632
25	0.5	0.492	0.585	0.488	0.632	0.485	0.579	0.271
26	0.433	0.403	0.484	0.54	0.39	0.34	0.533	0.539
27	0.504	0.548	0.447	0.395	0.443	0.458	0.419	0.432
28	0.25	0.395	0.342	0.565	0.574	0.659	0.383	0.629
29	0.522	0.462	0.349	0.332	0.43	0.431	0.565	0.401
30	0.603	0.567	0.653	0.65	0.511	0.396	0.46	0.264
31	0.366	0.575	0.615	0.623	0.346	0.464	0.455	0.437
32	0.468	0.521	0.496	0.512	0.33	0.453	0.436	0.438
33	0.466	0.53	0.426	0.326	0.53	0.425	0.396	0.671
34	0.343	0.44	0.623	0.395	0.531	0.456	0.359	0.533
35	0.436	0.568	0.421	0.727	0.473	0.59	0.593	0.453
36	0.487	0.554	0.626	0.506	0.302	0.719	0.413	0.452
37	0.437	0.498	0.515	0.505	0.486	0.549	0.579	0.414
38	0.423	0.623	0.388	0.474	0.603	0.607	0.632	0.51
39	0.65	0.472	0.544	0.545	0.494	0.433	0.401	0.343
40	0.521	0.352	0.573	0.445	0.395	0.408	0.416	0.355
41	0.485	0.522	0.416	0.621	0.509	0.502	0.43	0.451
42	0.448	0.394	0.509	0.339	0.483	0.43	0.512	0.469
43	0.462	0.511	0.471	0.572	0.507	0.399	0.322	0.431
44	0.593	0.436	0.386	0.563	0.439	0.404	0.435	0.431
45	0.332	0.66	0.421	0.58	0.509	0.535	0.409	0.494
46	0.471	0.538	0.473	0.469	0.507	0.381	0.479	0.502
47	0.477	0.343	0.563	0.529	0.617	0.479	0.33	0.495
48	0.576	0.449	0.425	0.415	0.415	0.488	0.658	0.484
49	0.495	0.355	0.524	0.558	0.495	0.46	0.5	0.628
50	0.547	0.539	0.457	0.388	0.573	0.487	0.506	0.465

Table S9. Detrended Fluctuation Analysis (DFA) scaling exponent γ values for the 400 shuffled amoeba trajectories.

Table S10

Cell type	Scenario	RMSFα	Correlation time	MSDβ	DFAγ	ApEn	Intensity of Response	Directionality Ratio	Average Speed
Cells	Sc1-Sc2	0.007	0.992	0.078	0.717	0.003	10^{-4}	10^{-5}	0.022
	Sc1-Sc3	0.058	0.261	0.791	0.828	0.319	0.556	0.627	0.791
	Sc1-Sc4	0.528	0.857	0.013	0.026	0.281	0.002	0.082	10^{-5}
	Sc2-Sc3	0.229	0.22	0.02	0.426	10^{-6}	0.006	10^{-6}	0.147
	Sc2-Sc4	0.012	0.978	10^{-6}	0.028	10^{-5}	10^{-9}	10^{-9}	10^{-9}
	Sc3-Sc4	0.127	0.16	0.008	0.022	0.692	10^{-4}	0.149	10^{-5}
	All	0.013	0.493	10^{-4}	0.048	10^{-5}	10^{-9}	10^{-9}	10^{-8}
Cytoplasts	Sc1-Sc2	0.77	0.152	0.196	0.024	10^{-4}	0.333	0.007	0.319
	Sc1-Sc3	0.807	0.931	0.038	0.165	0.068	0.652	0.009	0.662
	Sc1-Sc4	0.272	0.653	0.546	0.343	0.033	0.055	0.115	0.035
	Sc2-Sc3	0.92	0.099	0.333	0.354	0.051	0.497	0.828	0.169
	Sc2-Sc4	0.41	0.034	0.309	0.125	0.488	0.281	0.35	0.001
	Sc3-Sc4	0.497	0.678	0.071	0.637	0.467	0.073	0.224	0.065
	All	0.738	0.183	0.122	0.126	0.007	0.182	0.023	0.013
Cells Vs. Cytoplasts	Sc1	0.068	0.074	0.02	0.112	10^{-4}	10^{-4}	0.033	10^{-4}
	Sc2	10^{-7}	0.445	10^{-4}	0.251	10^{-6}	10^{-9}	10^{-5}	10^{-9}
	Sc3	10^{-5}	0.238	0.749	0.622	0.088	10^{-6}	0.309	0.001
	Sc4	0.035	0.004	0.398	0.112	0.588	0.488	0.368	0.017
	Sc1-Sc2	0.111	0.582	0.163	0.488	0.316	0.001	0.743	10^{-6}
	Sc1-Sc3	0.088	0.039	0.603	0.801	0.014	10^{-4}	0.565	0.002
	Sc1-Sc4	0.333	0.019	0.046	0.414	0.143	0.007	0.642	0.12
	Sc2-Sc3	10^{-6}	0.035	0.013	0.942	10^{-10}	10^{-11}	10^{-4}	10^{-6}
	Sc2-Sc4	10^{-5}	0.019	10^{-5}	0.395	10^{-5}	10^{-8}	10^{-6}	0.001
	Sc3-Sc4	10^{-4}	0.122	0.04	0.281	0.391	0.002	0.712	0.123
	All	10^{-11}	0.001	0.011	0.993	10^{-7}	10^{-12}	0.047	10^{-8}

Table S10. Kruskal-Wallis and Wilcoxon comparison p-values to evaluate inter-cell-type and inter-scenario variability in the kinematic and systemic properties of the 400 experimental amoeba trajectories.

Table S11. Values (Mean/STD) of the non-linear metrics and the duration of the correlation regimes for the 280 enucleated cell trajectories obtained during the first 15 minutes after the recovery time. Trajectories were studied using a 5-minute sliding window (a moving time frame applied sequentially), advanced in 1-minute steps.

Time Window\Metric	RMSFα	Time Dependency (min)	MSDβ	DFAγ	Approximate Entropy (ApEn)
0’ – 5’	0.741/0.100	1.488/0.524	1.854/0.126	1.644/0.291	0.018/0.016
1’ – 6’	0.728/0.100	1.482/0.523	1.857/0.120	1.618/0.346	0.017/0.016
2’ – 7’	0.731/0.103	1.506/0.526	1.872/0.107	1.661/0.315	0.015/0.014
3’ – 8’	0.728/0.118	1.476/0.514	1.883/0.104	1.696/0.302	0.013/0.011
4’ – 9’	0.719/0.108	1.420/0.502	1.888/0.094	1.706/0.280	0.013/0.011
5’ – 10’	0.714/0.123	1.433/0.505	1.892/0.098	1.699/0.297	0.013/0.015
6’ – 11’	0.722/0.130	1.441/0.515	1.898/0.093	1.718/0.293	0.013/0.012
7’ – 12’	0.727/0.122	1.490/0.517	1.899/0.095	1.724/0.329	0.013/0.012
8’ – 13’	0.727/0.129	1.460/0.520	1.894/0.095	1.709/0.312	0.013/0.011
9’ – 14’	0.737/0.123	1.505/0.527	1.891/0.094	1.679/0.341	0.013/0.012
10’ – 15’	0.731/0.116	1.474/0.514	1.886/0.102	1.687/0.313	0.013/0.010
STD	0.008	0.029	0.016	0.033	0.002

Table S12. Values of the non-linear metrics and the correlation times for the 80 new enucleated cells included in the quantitative analysis (the values of the remaining 200 enucleated cell trajectories are in Tables S1, S4, S6, and S8).

Cell #	RMSFα	MSDβ	DFAγ	ApEn	Cell #	RMSFα	MSDβ	DFAγ	ApEn
**1**	0.890	1.735	1.399	0.007	**41**	0.797	1.990	1.947	0.005
**2**	0.777	1.973	1.777	0.006	**42**	0.789	1.925	1.636	0.018
**3**	0.772	1.744	1.500	0.007	**43**	0.705	1.869	1.851	0.005
**4**	0.818	1.428	1.437	0.004	**44**	0.834	1.929	1.765	0.004
**5**	0.823	1.991	1.854	0.002	**45**	0.768	0.880	1.161	0.018
**6**	0.710	1.706	1.737	0.003	**46**	0.757	1.948	1.865	0.003
**7**	0.764	1.816	1.449	0.005	**47**	0.825	2.000	1.921	0.003
**8**	0.793	1.684	1.345	0.005	**48**	0.759	1.740	1.715	0.004
**9**	0.767	1.832	1.774	0.002	**49**	0.659	1.975	1.856	0.002
**10**	0.829	1.956	1.800	0.003	**50**	0.804	1.976	1.760	0.005
**11**	0.735	1.820	1.769	0.003	**51**	0.719	1.707	1.542	0.009
**12**	0.774	1.930	1.751	0.004	**52**	0.752	1.922	1.738	0.003
**13**	0.807	1.903	1.850	0.002	**53**	0.611	1.831	1.783	0.004
**14**	0.797	1.878	1.881	0.005	**54**	0.663	1.912	1.837	0.007
**15**	0.706	1.947	1.829	0.005	**55**	0.691	1.963	1.874	0.008
**16**	0.614	1.694	1.813	0.011	**56**	0.774	1.940	1.833	0.002
**17**	0.732	1.633	1.500	0.008	**57**	0.806	1.722	1.572	0.003
**18**	0.790	1.680	1.662	0.010	**58**	0.835	1.956	1.841	0.002
**19**	0.704	1.885	1.857	0.006	**59**	0.791	1.981	1.869	0.002
**20**	0.763	1.956	1.872	0.003	**60**	0.760	1.961	1.903	0.003
**21**	0.811	1.695	1.185	0.018	**61**	0.770	1.881	1.844	0.002
**22**	0.762	1.738	1.167	0.008	**62**	0.822	1.922	1.584	0.003
**23**	0.814	1.853	1.761	0.005	**63**	0.730	1.746	1.726	0.005
**24**	0.863	1.954	1.508	0.004	**64**	0.778	1.934	1.824	0.002
**25**	0.791	1.919	1.768	0.004	**65**	0.805	1.872	1.760	0.004
**26**	0.829	1.751	1.711	0.006	**66**	0.834	1.542	1.407	0.005
**27**	0.751	1.927	1.861	0.004	**67**	0.705	1.871	1.772	0.004
**28**	0.791	1.825	1.611	0.004	**68**	0.763	1.684	1.354	0.006
**29**	0.729	1.715	1.504	0.007	**69**	0.667	1.752	1.659	0.004
**30**	0.809	1.903	1.405	0.005	**70**	0.679	1.557	1.423	0.009
**31**	0.748	1.952	1.809	0.003	**71**	0.757	1.861	1.795	0.003
**32**	0.766	1.755	1.643	0.008	**72**	0.736	1.845	1.802	0.002
**33**	0.641	1.996	1.926	0.007	**73**	0.665	1.983	1.874	0.004
**34**	0.838	1.785	1.577	0.006	**74**	0.734	1.939	1.832	0.005
**35**	0.834	1.943	1.755	0.005	**75**	0.735	1.519	1.285	0.010
**36**	0.669	1.985	1.858	0.004	**76**	0.738	1.796	1.846	0.004
**37**	0.741	1.891	1.763	0.005	**77**	0.929	1.930	1.634	0.012
**38**	0.778	1.926	1.818	0.006	**78**	0.791	1.942	1.815	0.004
**39**	0.748	1.815	1.284	0.017	**79**	0.677	1.935	1.844	0.002
**40**	0.890	1.735	1.399	0.007	**80**	0.756	1.686	1.667	0.007

Table S13. Comparison between the migratory properties of intact non-enucleated cells, enucleated cells, and non-enucleated cells with a small portion of their cytoplasm removed. Median and interquartile range (IQR) of the eight calculated metrics for each cell type are shown.

**Metric**	**Intact non-enucleated cells (Median/IQR, see Table 1 of the manuscript)**	**Enucleated cells (Median/IQR, see Table 1 of the manuscript)**	**Non-enucleated cells with a small portion of their cytoplasm removed (Median/IQR, see Tables S14-15 of the Supporting Information)**
RMSF α	0.74/0.07	0.77/0.07	0.77/0.05
Correlation time (min)	10.42/8.33	8.33/5.21	9.38/4.69
MSD β	1.93/0.11	1.91/0.11	1.90/0.14
DFA γ	1.82/0.08	1.82/0.09	1.82/0.11
Aproximate Entropy	0.002/0.001	0.003/0.001	0.003/0.001
Intensity of the Response (mm)	4.31/3.04	2.47/2.42	2.79/3.51
Directionality Ratio	0.62/0.21	0.60/0.28	0.61/0.25
Average Speed (µm/s)	1.70/0.78	1.34/0.84	1.42/0.82

Table S14. Values for the non-linear metrics of the 25 non-enucleated cells with a small portion of their cytoplasm removed.

#	RMSF α				Correlation time (min)				MSD β				ApEn				DFA γ			
	Sc1	Sc2	Sc3	Sc4	Sc1	Sc2	Sc3	Sc4	Sc1	Sc2	Sc3	Sc4	Sc1	Sc2	Sc3	Sc4	Sc1	Sc2	Sc3	Sc4
1	0.799	0.745	0.761	0.693	5.208	9.375	6.250	16.667	2.003	1.899	2.004	1.956	0.003	0.003	0.003	0.001	1.413	1.869	1.842	1.641
2	0.695	0.773	0.733	0.659	9.375	16.667	8.333	5.208	1.977	1.979	1.889	1.933	0.003	0.001	0.003	0.002	1.806	1.830	1.814	1.841
3	0.745	0.803	0.769	0.738	7.292	10.417	8.333	11.458	1.570	1.761	1.619	1.938	0.003	0.003	0.002	0.002	1.835	1.830	1.815	1.874
4	0.766	0.762	0.750	0.795	5.208	9.375	7.292	4.167	1.941	1.871	1.945	1.952	0.002	0.002	0.002	0.001	1.863	1.874	1.868	1.641
5	0.808	0.774	0.784	0.718	3.125	7.292	10.417	7.292	1.962	1.979	1.986	1.730	0.002	0.002	0.002	0.001	1.753	1.583	1.470	1.891
6	0.766	0.807	0.753	0.744	9.375	14.583	16.667	4.167	2.007	1.974	1.889	1.838	0.003	0.010	0.002	0.002	1.768	1.831	1.777	1.827
7	0.813	0.725	0.785	0.784	14.583	10.417	16.667	8.333	1.313	1.936	1.807	1.627	0.003	0.001	0.002	0.006	1.779	1.852	1.667	1.873
8	0.807	0.851	0.794	0.782	15.625	9.375	5.208	13.542	1.962	1.902	1.905	1.926	0.005	0.002	0.002	0.006	1.787	1.823	1.853	1.887
9	0.695	0.680	0.813	0.738	14.583	10.417	7.292	6.250	1.727	1.955	1.814	1.635	0.003	0.002	0.003	0.002	1.809	1.905	1.667	1.870
10	0.766	0.826	0.752	0.721	5.208	17.708	8.333	10.417	1.890	1.979	1.979	2.013	0.002	0.002	0.005	0.002	1.743	1.836	1.899	1.723
11	0.787	0.757	0.799	0.754	2.083	7.292	8.333	9.375	1.975	1.863	1.881	1.881	0.003	0.003	0.004	0.002	1.863	1.544	1.908	1.723
12	0.763	0.742	0.720	0.738	8.333	7.292	10.417	13.542	1.664	1.913	1.939	1.819	0.002	0.004	0.002	0.003	1.779	1.836	1.825	1.848
13	0.808	0.784	0.769	0.764	11.458	10.417	6.250	15.625	1.758	1.822	1.924	1.952	0.005	0.001	0.003	0.002	1.835	1.862	1.850	1.843
14	0.799	0.826	0.752	0.785	17.708	17.708	10.417	9.375	1.941	1.903	1.985	1.730	0.009	0.003	0.002	0.003	1.759	1.874	1.759	1.811
15	0.746	0.851	0.814	0.826	9.375	12.500	14.583	7.292	1.739	1.903	1.857	1.824	0.009	0.003	0.004	0.003	1.863	1.836	1.470	1.887
16	0.695	0.680	0.761	0.780	6.250	8.333	7.292	7.292	1.570	1.972	1.900	1.800	0.002	0.002	0.003	0.001	1.854	1.791	1.904	1.600
17	0.798	0.807	0.766	0.738	6.250	10.417	2.083	5.208	1.957	1.972	1.884	1.889	0.003	0.010	0.003	0.001	1.818	1.807	1.853	1.800
18	0.807	0.842	0.750	0.769	6.250	9.375	4.167	8.333	1.977	1.967	1.857	1.822	0.012	0.010	0.002	0.003	1.863	1.544	1.586	1.772
19	0.783	0.725	0.765	0.835	11.458	10.417	10.417	6.250	1.897	1.872	2.001	1.938	0.002	0.002	0.002	0.004	1.639	1.544	1.815	1.674
20	0.807	0.806	0.766	0.738	15.625	14.583	14.583	5.208	1.562	1.929	1.800	1.952	0.003	0.002	0.003	0.004	1.499	1.848	1.842	1.788
21	0.759	0.784	0.776	0.783	11.458	12.500	6.250	5.208	1.797	1.795	1.913	1.942	0.002	0.002	0.004	0.002	1.788	1.864	1.832	1.752
22	0.747	0.784	0.740	0.856	14.583	9.375	8.333	10.417	1.975	1.795	1.881	1.887	0.004	0.003	0.001	0.001	1.484	1.436	1.777	1.835
23	0.762	0.709	0.825	0.799	5.208	8.333	14.583	9.375	1.791	1.602	1.881	1.800	0.002	0.004	0.004	0.003	1.737	1.815	1.739	1.891
24	0.736	0.757	0.833	0.718	4.167	7.292	7.292	16.667	1.375	1.892	1.855	1.943	0.010	0.002	0.005	0.002	1.372	1.945	1.669	1.600
25	0.763	0.826	0.778	0.795	14.583	7.292	7.292	5.208	1.903	1.936	1.985	1.932	0.002	0.003	0.004	0.002	1.718	1.817	1.822	1.762

Table S15. Values for the cytokinematic metrics of the 25 non-enucleated cells with a small portion of their cytoplasm removed.

#	Intensity of the response (mm)				Directionality Ratio				Average speed (µm/s)			
	Sc1	Sc2	Sc3	Sc4	Sc1	Sc2	Sc3	Sc4	Sc1	Sc2	Sc3	Sc4
1	5.675	1.337	3.177	2.509	0.849	0.509	0.646	0.583	1.085	1.201	0.398	1.568
2	2.609	2.106	4.466	6.530	0.547	0.575	0.837	0.807	2.083	1.848	1.584	1.611
3	4.776	7.753	2.813	9.346	0.249	0.620	0.739	0.678	1.024	0.410	2.498	2.441
4	4.797	1.379	2.614	4.987	0.546	0.671	0.863	0.246	1.007	1.935	0.857	1.322
5	3.139	5.098	2.775	1.473	0.341	0.559	0.756	0.358	1.049	1.225	1.354	1.673
6	0.338	2.144	5.774	4.997	0.286	0.418	0.350	0.422	1.023	2.330	1.019	1.356
7	4.033	3.220	4.876	1.415	0.830	0.671	0.793	0.078	2.223	0.746	1.462	0.514
8	0.704	3.240	4.897	2.350	0.295	0.733	0.654	0.285	1.425	1.288	1.889	1.612
9	3.618	3.049	1.122	0.258	0.547	0.418	0.802	0.628	2.135	1.553	1.414	1.672
10	1.271	0.683	0.831	2.759	0.745	0.575	0.751	0.558	0.943	1.289	1.698	2.807
11	5.892	3.501	6.830	4.168	0.724	0.509	0.247	0.560	2.090	0.949	1.642	0.723
12	0.884	2.072	2.300	3.548	0.455	0.607	0.499	0.664	1.360	1.752	1.431	1.318
13	6.670	1.777	1.043	6.541	0.707	0.758	0.749	0.358	1.751	0.958	1.463	1.274
14	2.598	5.264	2.535	2.332	0.743	0.652	0.739	0.078	1.428	1.270	2.193	0.469
15	0.863	0.472	0.131	0.903	0.227	0.541	0.756	0.628	1.820	2.992	2.987	0.701
16	0.267	5.312	0.593	4.801	0.581	0.764	0.498	0.622	3.364	1.888	1.356	1.631
17	3.220	0.715	7.721	3.513	0.258	0.764	0.455	0.297	1.100	1.224	0.918	1.242
18	1.925	1.394	4.866	4.826	0.515	0.573	0.630	0.372	1.750	0.974	1.440	0.923
19	0.226	5.410	5.796	6.223	0.476	0.706	0.564	0.737	2.248	0.748	1.857	2.443
20	3.209	2.575	0.875	2.364	0.825	0.617	0.766	0.602	1.892	1.325	1.134	1.759
21	1.770	1.637	4.456	4.178	0.627	0.819	0.393	0.678	0.428	1.934	1.257	2.085
22	4.786	5.244	1.133	4.816	0.849	0.541	0.837	0.583	0.914	0.755	1.249	1.491
23	1.031	5.292	2.364	2.499	0.547	0.594	0.564	0.777	1.071	1.414	1.355	2.503
24	0.256	5.388	0.503	6.253	0.428	0.746	0.646	0.418	2.503	1.889	1.439	0.722
25	0.215	3.081	5.785	0.893	0.825	0.712	0.612	0.704	0.961	0.783	1.850	2.446

SI References

1. J. W. Gibbs, *Elementary principles in statistical mechanics: developed with especial reference to the rational foundations of thermodynamics* (Charles Scribner's sons, 1902).

2. A. Einstein, Zum gegenwärtigen Stand des Strahlungsproblems. *Phys. Z.* **10**, 185–193 (1909).

3. P. Ch. Ivanov, *et al.*, Multifractality in human heartbeat dynamics. *Nature* **399**, 461–465 (1999).

4. P. Ch. Ivanov, *et al.*, From 1/f noise to multifractal cascades in heartbeat dynamics. *Chaos Interdiscip. J. Nonlinear Sci.* **11**, 641–652 (2001).

5. G. M. Viswanathan, *et al.*, Lévy flight search patterns of wandering albatrosses. *Nature* **381**, 413–415 (1996).

6. A. Einstein, Über die von der molekularkinetischen Theorie der Wärme geforderte Bewegung von in ruhenden Flüssigkeiten suspendierten Teilchen. *Ann. Phys.* **322**, 549–560 (1905).

7. Z. Long, *et al.*, Microfluidic chemostat for measuring single cell dynamics in bacteria. *Lab. Chip* **13**, 947–954 (2013).

8. R. Gorelik, A. Gautreau, Quantitative and unbiased analysis of directional persistence in cell migration. *Nat. Protoc.* **9**, 1931–1943 (2014).

9. G. M. Viswanathan, E. P. Raposo, M. G. E. da Luz, Lévy flights and superdiffusion in the context of biological encounters and random searches. *Phys. Life Rev.* **5**, 133–150 (2008).

10. C.-K. Peng, *et al.*, Mosaic organization of DNA nucleotides. *Phys Rev E* **49**, 1685–1689 (1994).

11. A. L. Goldberger, *et al.*, Fractal dynamics in physiology: Alterations with disease and aging. *Proc. Natl. Acad. Sci.* **99**, 2466–2472 (2002).

12. R. Hardstone, *et al.*, Detrended Fluctuation Analysis: A Scale-Free View on Neuronal Oscillations. *Front. Physiol.* **3** (2012).

13. S. M. Pincus, I. M. Gladstone, R. A. Ehrenkranz, A regularity statistic for medical data analysis. *J. Clin. Monit.* **7**, 335–345 (1991).

14. L. Cao, Practical method for determining the minimum embedding dimension of a scalar time series. *Phys. Nonlinear Phenom.* **110**, 43–50 (1997).
